# Knowledge-Augmented Large Language Model for Multimodal Electronic Health Record–Based Risk Prediction: Development and Validation Study

**DOI:** 10.2196/88356

**Published:** 2026-06-12

**Authors:** Rituparna Datta, Jiaming Cui, Zihan Guan, Vishal Reddy, Joshua Eby, Gregory R Madden, Rupesh Silwal, Anil Vullikanti

**Affiliations:** 1 University of Virginia Charlottesville, VA United States; 2 Virginia Tech Blacksburg, VA United States; 3 School of Medicine University of Virginia Charlottesville, VA United States; 4 Department of Medicine University of Virginia Charlottesville, VA United States

**Keywords:** biomedical knowledge graphs, clinical risk prediction, EHR, electronic health records, knowledge-augmented reasoning, large language models, machine learning, multimodal data integration

## Abstract

**Background:**

Accurate clinical outcome prediction using electronic health records (EHRs) is crucial for patient care and resource allocation. EHRs include both structured data and rich, unstructured clinical notes. However, prior machine learning methods struggle with the multimodality, long context of notes, and severe class imbalance in clinical tasks.

**Objective:**

This study aimed to introduce and evaluate KAMELEON (Knowledge-Augmented Multimodal EHR Learning for Outcome Prediction), a unified, 2-stage hybrid framework that integrates diverse EHR modalities and external biomedical knowledge to enhance clinical risk prediction.

**Methods:**

This study used the publicly available, deidentified Medical Information Mart for Intensive Care-III dataset, which includes structured and unstructured data for over 40,000 intensive care unit patients. The 2 tasks studied were 30-day readmission (403/10,031, 4% positive rate) and in-hospital mortality prediction (2423/17,903, 13% positive rate). Train-test splits were patient-disjoint (80:20). Performance was evaluated against general and medical large language models (LLMs) and structured baselines. Key metrics included the area under the receiver operating characteristic curve (AUROC), area under the precision-recall curve (AUPRC), and macro *F*_1_-score.

**Results:**

The KAMELEON framework consistently outperformed all existing baselines. 30-day readmission: the KAMELEON-balanced random forests model achieved an AUROC of 0.85 and a sensitivity (recall) of 0.79. Ablation analysis shows the critical role of the LLM-generated reasoning, with its removal causing the AUROC to drop from 0.85 to 0.7 and sensitivity to fall by over 80%. In-hospital mortality: the KAMELEON-extreme gradient boosting model achieved an AUROC of 0.92 and an AUPRC of 0.650. Unstructured-only models showed limited ability to discern mortality, with AUROC values near chance (around 0.51-0.53).

**Conclusions:**

To our knowledge, KAMELEON represents one of the first systematic frameworks to enhance LLMs for health care prediction through graph-guided knowledge retrieval combined with structured machine learning. The framework demonstrates superior performance across both prediction tasks, highlighting the synergistic value of combining diverse data modalities and LLM reasoning for robust clinical risk estimation.

## Introduction

Appropriate use of clinical prediction tools for early identification of high-risk patients for different conditions allows for clinical decision-making, timely interventions, escalation of care, intensive monitoring, and identification of gaps in outpatient management [1-5]. For instance, readmission within a short period is a priority under many regulatory frameworks and value-based care models, where high readmission rates may lead to financial penalties [6,7]. Therefore, effective models of short-term risk prediction (eg, 30 days) can guide targeted interventions, including more detailed discharge instructions, closer postdischarge monitoring, or referrals to transitional care programs. While traditional prediction tools relied on simple statistical models, for example, regression and decision trees [8,9], for risk assessment, more complex machine learning (ML) methods are increasingly applied to clinical prediction tasks [10-14]. In this work, we focus on two commonly studied clinical problems: (1) 30-day readmission prediction, which determines whether a patient will be readmitted to the hospital within 30 days after discharge, and (2) mortality prediction, which determines the patient’s in-hospital mortality status.

There has been a lot of work on developing diverse kinds of ML methods for these problems using electronic health record (EHR) data, which contain rich information on patient health [1,8,10-18]. Most of this work focuses on structured EHR data, which includes admission/discharge information, procedures and interventions, medications, laboratory orders and results, billing codes (eg, *ICD* [*International Classification of Diseases*] and Current Procedural Terminology), and physiological time-series (eg, vital signs). While imaging data and clinical documentation (such as progress notes or discharge summaries) represent unstructured data sources, they have been underused in prior clinical prediction models or processed in overly simplified ways (eg, bag-of-words representations). Unstructured data from clinical notes have also been used in a fairly simple manner, such as bag of words or term frequency-inverse document frequency representations [19,20], to facilitate the use of conventional ML methods for clinical tasks. Clinical notes are complex and poorly structured, which limits their use in clinical informatics tasks, even when using advanced natural language processing techniques. While large language models (LLMs) offer a powerful means to process such notes, especially when combined with large biomedical datasets to capture richer semantics beyond keywords and embeddings [15,21-23], they still face significant limitations, such as hallucinations, factual inaccuracies, and inadequate domain grounding [15,21]. For instance, models such as Med-PaLM [23] exhibit strong language generation capabilities but frequently misinterpret similar-sounding medical terms. Recent approaches have attempted to enhance LLMs with structured knowledge via graph-based retrieval (eg, GraphRAG), but their performance remains limited due to a lack of explicit reasoning [14,24-27]. In recent work, Jiang et al [15] developed Knowledge Aware Reasoning-Enhanced HealthCare Prediction (KARE), a GraphRAG and context augmentation approach, for clinical prediction tasks, which address many challenges associated with using LLMs for clinical tasks on the Medical Information Mart for Intensive Care (MIMIC) dataset.

However, the performance of all prior methods remains limited because clinical tasks using EHRs present many nontrivial challenges: (1) multimodality of clinical data: the presence of both structured data and unstructured text requires methods capable of effectively learning from both modalities. (2) Long-context textual data: clinical text often contains a mix of specialized medical terminology and informal or colloquial expressions, making information retrieval challenging. (3) Severe class imbalance: prediction tasks are typically highly imbalanced. For example, only about 4% (403/10,031) of patients are readmitted within 30 days, resulting in heavily skewed training data.

**Figure 1:**
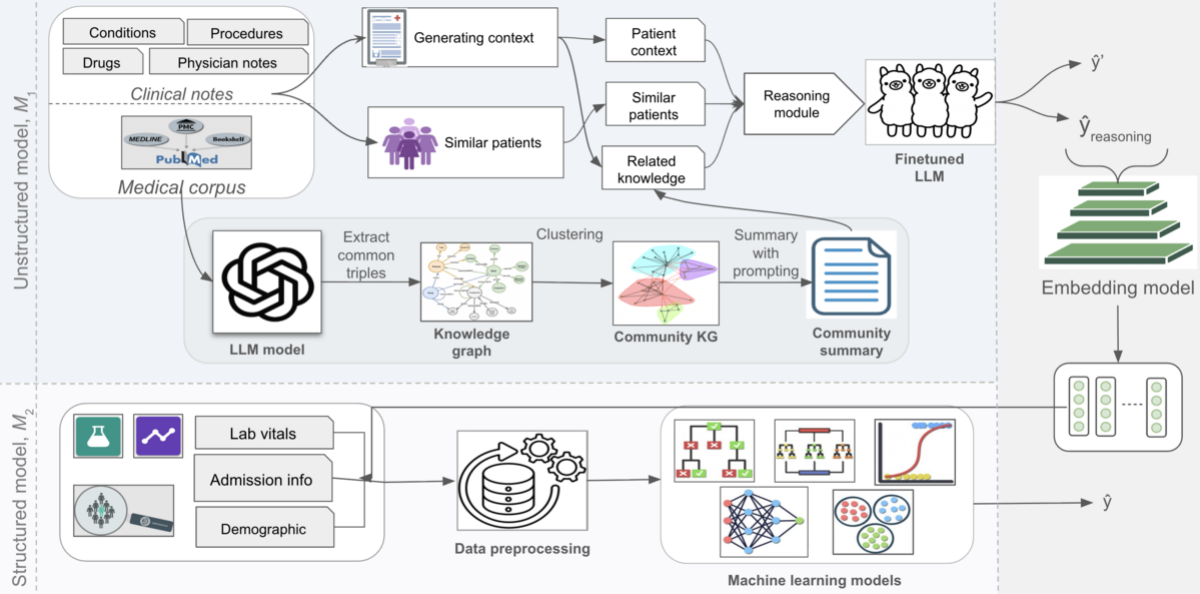
A 2-stage hybrid framework for predictive tasks, integrating structured and unstructured patient data with large language models (LLMs). Step 1 (M1) focuses on knowledge-enhanced context generation with an initial LLM output, while Step 2 (M2) integrates the fine-tuned LLM outputs with structured data by creating an embedding for final machine learning prediction. KG: knowledge graph.

Here, we develop a novel framework, KAMELEON (Knowledge-Augmented Multimodal EHR Learning for Outcome Prediction), that addresses the limitations of prior work by integrating multimodal EHR data (including structured clinical components and unstructured physician notes) and external biomedical knowledge. We refer to our approach as a “knowledge-augmented LLM,” as its predictions and reasoning are systematically enriched using external biomedical knowledge retrieved from a domain-specific knowledge graph (KG) constructed from the Unified Medical Language System (UMLS), PubMed abstracts, and LLM-generated entity-relation triples. KAMELEON consists of 2 components (as shown in Figure 1).

1. An unstructured model (*M*_1_) that processes clinical notes and retrieves relevant biomedical knowledge using a PubMed-derived graph and knowledge-augmented reasoning, and outputs a prediction for a patient, along with its reasoning; this extends the approach of Jiang et al [15]. Physician notes in EHRs can be lengthy and exceed the context window, and these are summarized using an LLM and used as context. To introduce domain-level medical knowledge, we build a biomedical KG by combining the UMLS [28], PubMed abstracts, and LLM-generated entity-relation triples. KG is partitioned into semantically coherent and well-connected clusters, and the textual summary generated by an LLM for the most relevant clusters for each patient cluster is used to enrich the context. Furthermore, labeled context is added by identifying semantically similar patient visits, which is used to fine-tune the LLM. Finally, *M*_1_ produces a prediction for the patient, along with a reasoning.
2. A structured model (*M*_2_), which extracts structured features from the patient’s EHR for the stay, including (1) static demographic and admission data, (2) time-varying vitals (which are normalized and summarized, when used as features), and (3) diagnoses, procedures, and medications. In addition, *M*_2_ includes the LLM’s prediction and its tokenized reasoning transformed into an embedding, as inputs. Finally, different kinds of standard ML methods are trained in *M*_2_ using these inputs. We first train *M*_1_ separately and use the LLM outputs to train *M*_2_.

We demonstrate the effectiveness of KAMELEON for the 30-day readmission risk and mortality prediction tasks, which have been studied extensively, both using MIMIC-III and other private EHRs from specific hospitals. We compare the performance with a number of structured ML and LLM baselines, with respect to multiple metrics, KAMELEON consistently outperforms all prior work on MIMIC-III datasets. It also shows clear gains over the strongest unstructured LLM baseline (LLaMA3-Med42-8B). The only other prior work that has similar performance for 30-day readmission [29] is on a Norwegian EHR dataset, which is significantly less imbalanced (5936/35,591, 16*.*7% readmission positive rate, instead of 403/10,031, 4% in the case of MIMIC-III; Table 1).

In summary, KAMELEON is the first systematic framework to enhance the power of LLMs for health care prediction tasks through graph-guided knowledge retrieval combined with structured ML methods. We expect this framework to be readily applicable to other clinical questions beyond those examined in this study.

**Table 1:** Dataset statistics for mortality and readmission prediction tasks. Positive denotes that the target outcome occurred.

Task and split			Samples, n		Positive, %
**In-hospital** **mortality**					
	Train	17,903		13*.*53	
	Test	3236		11*.*55	
**Readmission** **in** **30** **days**					
	Train	10,031		4*.*01	
	Test	2425		3*.*80	

## Methods

### Background

#### Clinical Datasets

MIMIC-III data used in this study include both structured and unstructured data collected across patient visits, capturing the patient’s condition over time.

- Structured data includes standardized fields such as laboratory results, vital signs, and demographic attributes (eg, age, sex, and ethnicity). These are typically numeric or categorical and readily usable for statistical modeling.
- Unstructured data consists of free-text clinical documentation such as physician notes, as well as patient conditions, diagnoses, and prescribed medications.

Additional datasets include PubMed and the UMLS.

#### Problem Statements

To demonstrate the effectiveness of our method, we study 2 popular clinical tasks: in-hospital mortality prediction and 30-day readmission prediction [1,15]. We define these problems formally after introducing some notation.

### Notation

#### Overview

We use *v_i_* to denote a hospital visit by a patient. For each visit *v_i_*, the patient is associated with a set of medical data, *D_i_* = *D_i_*^struct^ *∪ D_i_*^unstruct^, comprising both structured information *D_i_*^struct^ (eg, codes, vitals, and laboratory results) and unstructured information *D*^unstruct^ (eg, clinical free-text notes). Our goal is to build a model *f_θ_* that predicts a patient’s target status based on their historical visit information, specifically, *y*ˆ*_i_* = *f_θ_* (*D_i_*).

#### 30-Day Readmission Problem

The objective is to determine whether the patient is readmitted to the hospital within 30 days following discharge from visit *v_i_*. We define the readmission indicator *y^readm^*



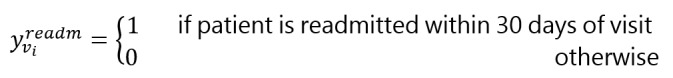



The goal is to develop a predictive model that estimates *y^readm^* using all structured and unstructured data from *v_i_*

#### Mortality Prediction Problem

Given the complete set of information for a visit *v_i_*, the objective is to determine the patient’s in-hospital mortality status, denoted as *y^mort^*, where:



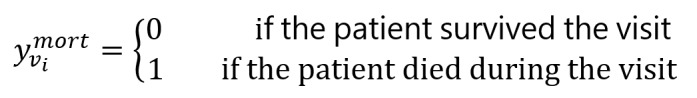



The goal is to develop a predictive model that accurately estimates *y^mort^* based on all available structured and unstructured clinical data associated with visit *v_i_*.

### KAMELEON Framework

#### Overview

We propose a hybrid framework, KAMELEON, that integrates multimodal EHR data, including structured clinical components and unstructured physician notes, and external biomedical knowledge to predict 2 key clinical outcomes: in-hospital mortality and 30-day readmission. As shown in Figure 1, KAMELEON consists of two components: (1) an unstructured encoder *M*_1_ that processes clinical notes and retrieves relevant biomedical knowledge using a PubMed-derived graph and knowledge-augmented reasoning, and outputs a prediction, along with its reasoning; and (2) a structured encoder *M*_2_ that combines multiple time-series corresponding to vitals and tabular datasets (laboratories, medications, etc), along with the outputs from *M*_1_ (ie, both the prediction and the embedding associated with the reasoning it produces) with static features for downstream prediction. The notations used in algorithms 1 and 2 are explained in Table 2.

**Table 2:** Summary of notation used in the framework.

Symbol	Description
_*X*_struct	Structured clinical features
_*X*_unstruct	Clinical free-text notes (eg, physician notes)
_*X*_demo	Demographic information
_*X*_sim	Embeddings of similar patient notes
*G* *,* *T*	Biomedical knowledge graph, triples
_*H*_text	Unstructured text embedding
_*H*_KG	Knowledge graph community summary embedding
_*H*_LLM reasoning	LLM^a^-generated reasoning with context
_*D*_LLM train/test	Augmented LLM training/test inputs
^*f*^LLM	Fine-tuned large language model
^*y*^^ˆ^reasoning^*,*^^*y*^^ˆ^^*′*^	LLM-generated textual reasoning, output label
^*f*^ML	Final machine learning classifier
_*H*_concat	Concatenated features for *f*_ML_
^*y*^^ˆ^task	Final binary classification output
^*L*^LLM	LLM fine-tuning loss
^*L*^task	Task-specific binary cross-entropy loss

^a^LLM: large language model.

#### Unstructured Data Encoder (M1)

##### Overview

For each hospital visit *v_i_*, we collect physician-authored clinical notes and extract entities like conditions, procedures, and medications. To enrich context, we use PubMed literature parsed into knowledge triples (subject-relation-object) via an LLM-based extraction pipeline. We retain only triples that appear across patient visits. These triples form a biomedical KG, serving as an auxiliary source to support LLM reasoning and diagnosis.

##### Generating Context

The first step of the framework is generating patient context. We use EHR data, including physicians’ notes, patient conditions, prescribed medications, and procedures in natural language. Since physician notes can be lengthy and exceed the context window of a small, locally fine-tuned LLM, we summarize them using an LLM. This approach addresses *Challenge 2 (Long Context)*.

##### KG Retrieval

**Figure 2:**
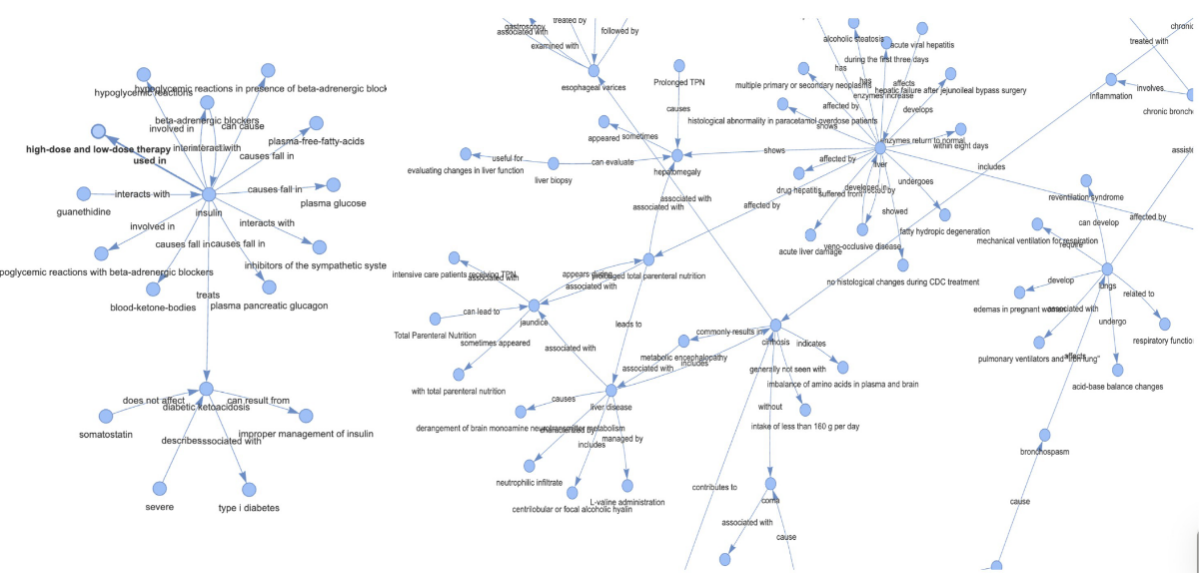
A partial snapshot of a knowledge graph built from PubMed data, filtered to include only patient-related concepts.

To introduce domain-level medical knowledge, we build a biomedical KG by combining the UMLS [28], PubMed abstracts, and LLM-generated entity-relation triples. UMLS provides standardized biomedical concepts and relationships, and the entity-relation triples are structured facts extracted by the LLM in the form (entity_1_*,* relation*,* entity_2_), capturing semantic connections. We apply the Leiden algorithm for community detection [30], which partitions the KG into semantically coherent and well-connected subgraphs. After clustering, we use a separate LLM to generate a textual summary for each cluster (Figure S8 in Multimedia Appendix 1). These summaries are produced by reasoning over the relationships among entities within each cluster, capturing the latent biomedical semantics encoded in the graph. Each summary serves as a high-level abstraction of the biomedical concepts and interactions within a subgraph. We embed these summaries using SentenceTransformer (MiniLM-L6-v2) [31] and retrieve the most relevant ones for each patient by computing semantic similarity with the embedded patient context. This process directly addresses *Challenge 3 (Specialized Medical Domains)* by enriching patient context with structured, domain-specific knowledge, improving the model’s understanding of specialized medical terminology. Figure 2 illustrates a partial biomedical KG constructed from PubMed data, which is used to retrieve domain-relevant knowledge associated with each patient’s clinical context.

##### Finding Similar Patients

We provide additional context by retrieving semantically similar patient visits using precomputed visit-level embeddings and a similarity index using the Facebook AI Similarity Search [32] library with inner-product search on L2-normalized embeddings, which effectively approximates cosine similarity. For each target patient visit, we retrieve the top 50 most similar patients while excluding self-matches and other visits from the same individual. Each retrieved patient is scored by similarity, and we filter them into positive and negative cohorts based on matching or nonmatching ground truth labels (eg, readmission vs no readmission). The final output includes the top-k positive and negative similar patients (with k=1,2). Unlike KARE [15], we also provide the physician notes of the retrieved similar patients, enabling the language model to leverage more clinical context when assessing patient risk. To prevent data leakage, we maintain patient-level separation between training and testing sets. The similarity index was constructed exclusively using training-set patient embeddings. During testing, each test patient retrieved similar cases only from the training-set hub.

##### Reasoning Module

In this module, we prepare inputs to fine-tune the LLM for clinical prediction. For each patient visit, we create a prompt with the patient’s context with the top-k most similar cases retrieved earlier. These similar cases guide the model by highlighting patterns in clinically comparable scenarios. We also add biomedical knowledge summaries from clustered subgraphs of a PubMed KG, providing literature-based context. Combining patient data, historical cases, and domain knowledge, we fine-tune the LLM to produce task-specific predictions with interpretable reasoning, supporting each outcome.

##### Fine-Tuning LLM

We fine-tune a *LLaMA-3 8B* model using the Unsloth framework [33,34], which enables memory-efficient training via 4-bit quantization and low-rank adaptation [35]. Prediction tasks are framed as instruction-following using Alpaca-style prompts with task description, patient context, and optional justification. Each prompt combines clinical notes, retrieved similar cases, and biomedical knowledge summaries. The model is trained via supervised learning to generate both predictions and reasoning. We use limited training steps with gradient accumulation and sequence lengths up to 8192 tokens. Unlike KARE, which trains larger models with higher compute, our method uses smaller, quantized models to reduce computational cost while maintaining interpretability and performance. Algorithm 1 outlines the training and inference procedures of *M*_1_. Additionally, an example prompt and its overall structure are provided in Multimedia Appendix 1.

#### Structured Data Encoder (M2)

##### Overview

We extract structured features from the patient’s visit history during each stay, including:

- Time-varying variables: we extract hourly vitals and diagnoses in MIMIC-III, including heart rate, systolic and diastolic blood pressure, mean blood pressure, oxygen saturation, Glasgow Coma Scale scores, glucose level, respiratory rate, temperature, weight, and pH.
- Static metadata: demographic and admission features include gender, ethnicity, admission type, location, insurance, language, and religion.
- Diagnoses, procedures, and medications in *ICD-9 (International Classification of Diseases, Ninth Revision)* codes: *ICD-9* codes, drug names are encoded via one-hot or counts. We compute binary indicators for key comorbidities (eg, sepsis, infection, organ failure, dementia, cancer, and diabetes).

While both structured and unstructured models use information about conditions, medications, and procedures, they access this information from different data modalities. *M*_1_ processes the clinical narrative and reasoning about these elements while *M*_2_ processes the structured codes and standardized entries.

##### Structured Data Preprocessing

The structured data are first transformed using a discretization step to enforce uniform temporal resolution and impute missing values. This is followed by normalization using precomputed mean and SD statistics over the continuous variables.

##### Incorporating LLM Output

To augment the structured input, we include the LLM’s prediction and its tokenized reasoning. For each patient visit, *M*_1_ generates (1) a prediction probability and (2) a textual reasoning explanation. The reasoning text is embedded using SentenceTransformer (all-MiniLM-L6-v2) [36], resulting in a 384-dimensional vector. This embedding, together with the scalar prediction probability, is concatenated with structured features and provided as input to the final classifier in *M*_2_.

##### Final Integration

The LLM-derived vector is merged with structured input features to create a unified representation, directly addressing *Challenge 1 (Multimodal Information)*. To reduce dimensionality and suppress noise from high-dimensional embeddings, we apply principal component analysis to the combined feature vector.

### Training

#### Overview

We follow a 2-stage training procedure. First, we fine-tune the unstructured text encoder *M*_1_ using instruction-style prompts built from clinical notes, retrieved similar cases, and external biomedical knowledge. After fine-tuning, we perform *final integration* and use the outputs of *M*_1_ as input features to train *M*_2_ for final prediction.

In our experiments, we benchmark several ML models for *M*_2_, such as KAMELEON-X, where X represents logistic regression, balanced random forests, long short-term memory, light gradient boosting machine (LightGBM), multilayer perceptron (MLP), or extreme gradient boosting (XGBoost), selected for its effectiveness in capturing clinical patterns. These models were selected based on class imbalance severity: the extreme imbalance in 30-day readmission favors BalancedRandomForest and regularized logistic regression, whereas the moderate imbalance in in-hospital mortality is better suited to gradient boosting methods that capture complex feature interactions; multiple architectures were evaluated before selecting the final task-specific model. For MLP, we use weighted binary cross-entropy loss



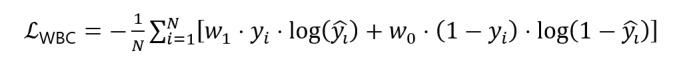



where *w*_1_ and *w*_0_ are the positive and negative class weights, respectively, used to address class imbalance by giving more emphasis to the minority class. We further use the synthetic minority over-sampling technique [37] to mitigate class imbalance, addressing *Challenge 4 (Highly imbalanced data)*.

In addition, for gradient boosting models, we use the scale_pos_weight parameter to up-weight minority samples during training. These complementary strategies—class-weighted loss, balanced bootstrapping, oversampling, and cost-sensitive boosting—ensure that minority-class examples are not overwhelmed by the dominant negative class and are consistent with our task-specific model selection based on imbalance severity.

For the KAMELEON-balanced random forests (BalancedRF) model, which is designed to handle class imbalance. Here, each decision tree is trained on a bootstrapped sample drawn by undersampling the majority class and combining it with all minority class examples, ensuring balanced class proportions at the tree level. Each split is chosen to minimize the *Gini impurity*:



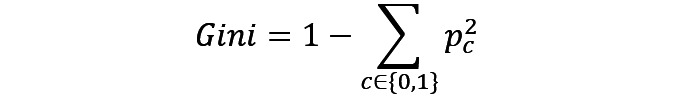



where *p_c_* denotes the proportion of class *c* in a given node. The balanced sampling overcomes the issue with the dominance of the majority class and improves sensitivity to rare outcomes. Final predictions are obtained by aggregating probabilities across trees.

The complete training and inference pipeline for *M*_2_ is outlined in algorithm 2.

#### Algorithm 1. Unstructured Data Encoder (M1)

Require: Training data: 
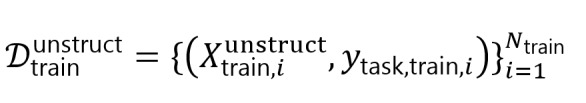


Require: Test data: 
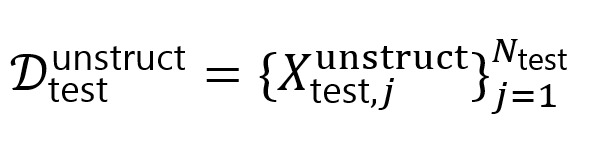


Ensure: Intermediate prediction 
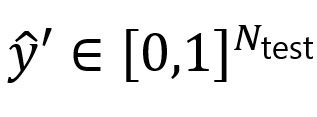
, Reasoning 
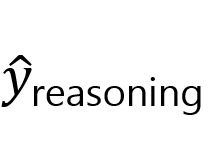


##### Phase 1: Preprocessing and LLM Input Preparation



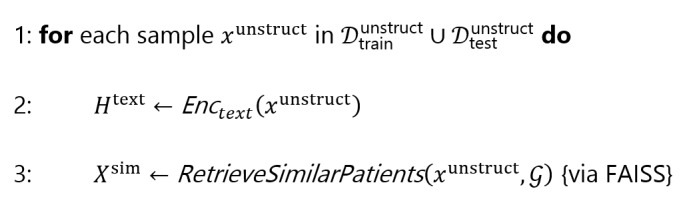





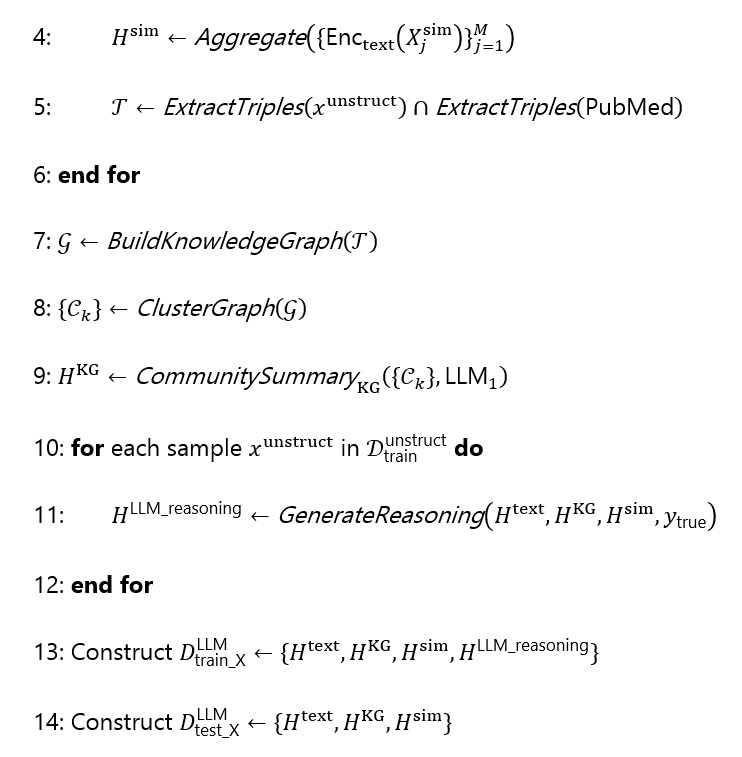



##### Phase 2: Fine-Tuning LLM Model (fLLM)



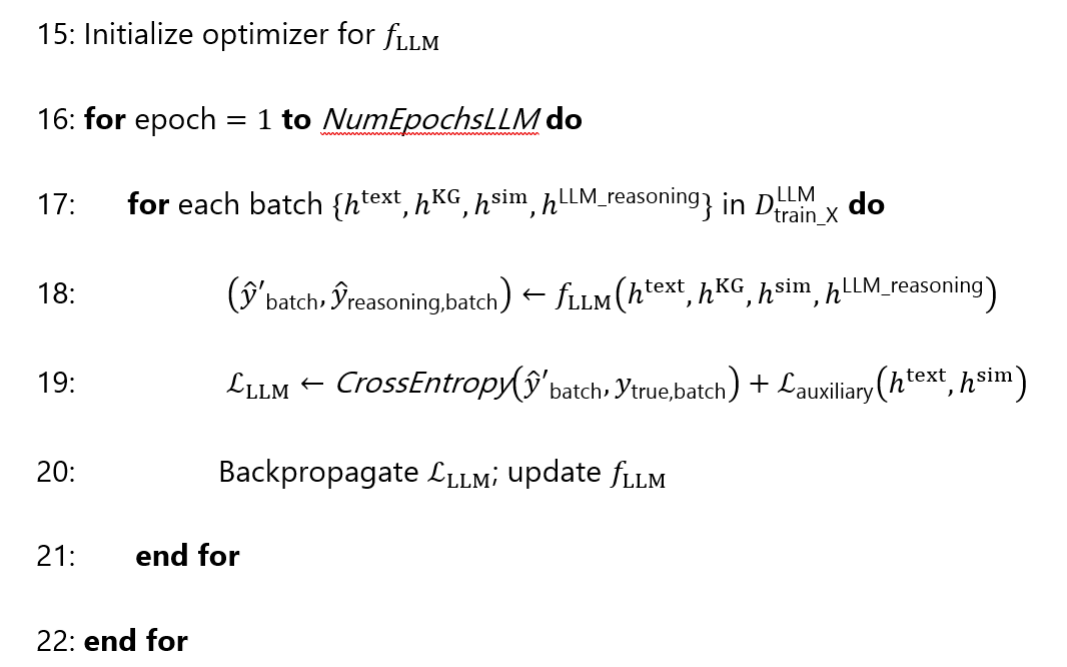



##### Phase 3: Inference With Fine-Tuned LLM



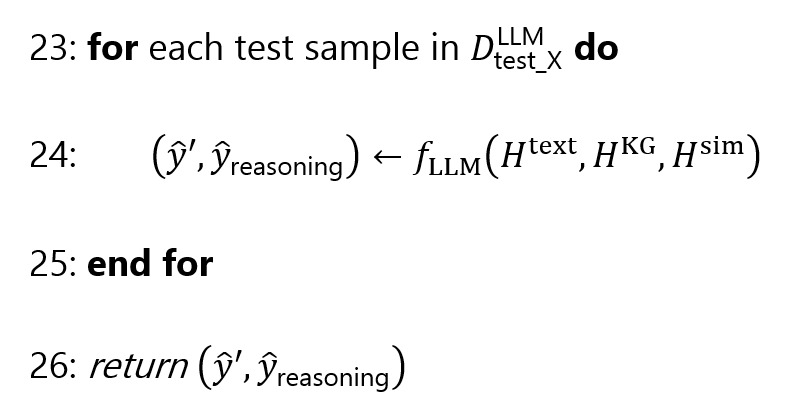



#### Algorithm 2. Structured Data Encoder (M2)

Require: Structured training data: 
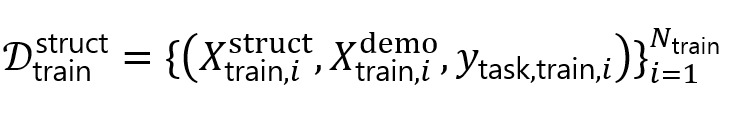


Require: Structured test data: 
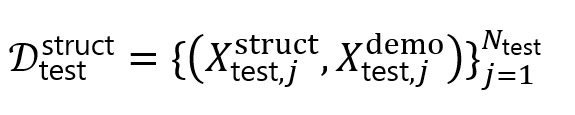


Require: From algorithm 1: 
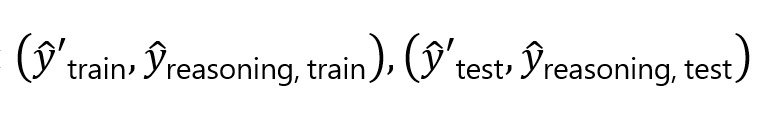


Ensure: Final prediction 
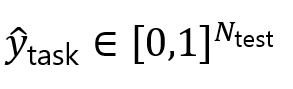


##### Phase 1: Training Final ML Model (fML)



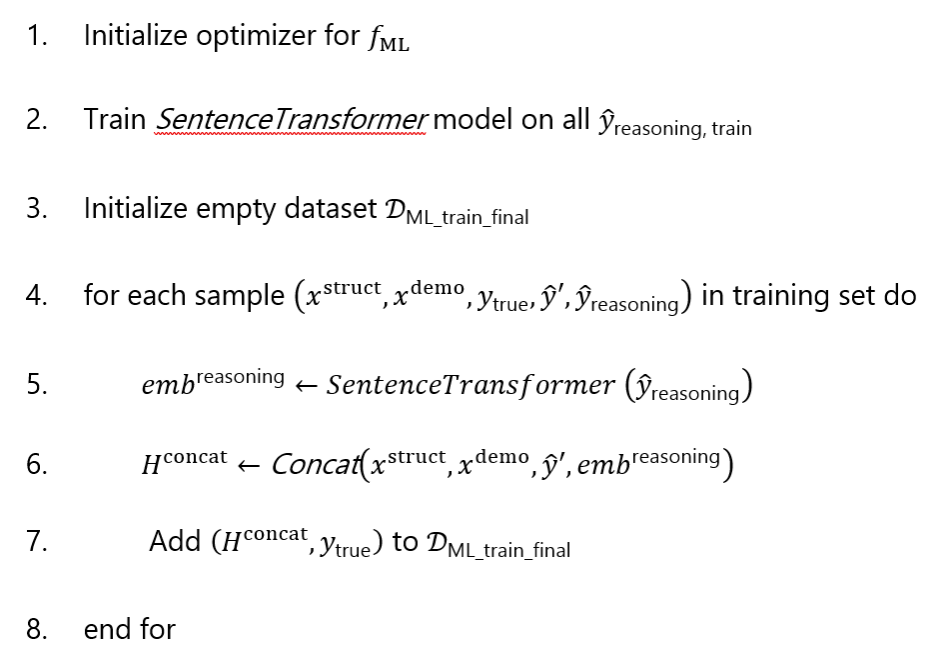





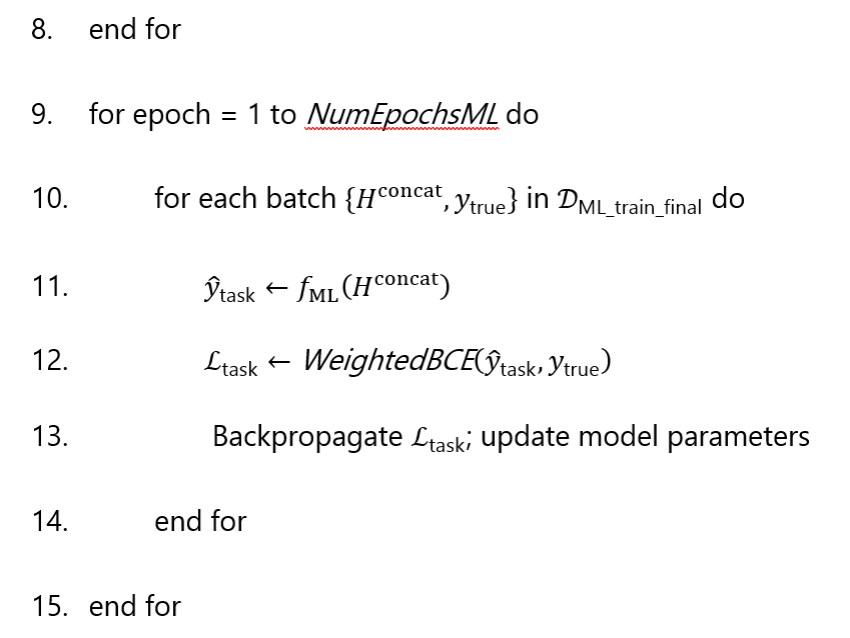



##### Phase 2: Inference on Test Set



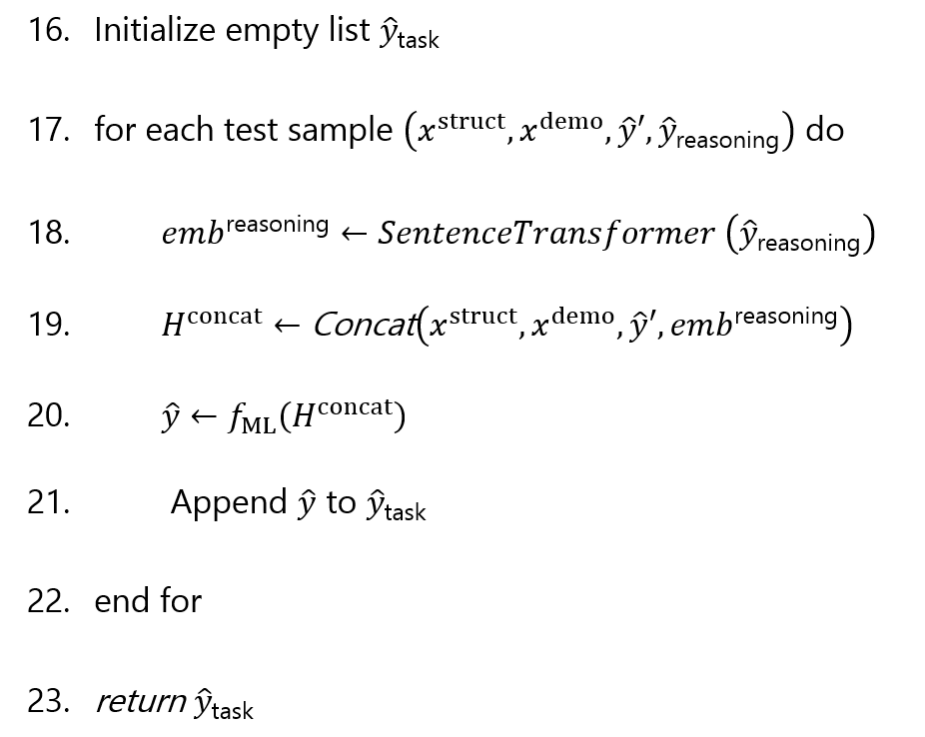



### Ethical Considerations

This study did not involve direct human participants. We used the MIMIC-III database, a publicly available, deidentified critical care dataset.

## Results

### Overview

We evaluate our model on the 2 clinical prediction tasks: in-hospital mortality and 30-day readmission. Our experiments compare performance against strong baselines, including general and medical LLMs, and traditional structured ML approaches. Given the severe class imbalance in 30-day readmission, we use balanced random forest (with internal bootstrap resampling) and regularized logistic regression (C = 1.0/0.01) to mitigate minority-class overfitting. In contrast, in-hospital mortality (2423/17,903, 13% positive rate) presents a moderate imbalance, for which gradient boosting methods (XGBoost and LightGBM) are better suited due to their ability to capture complex feature interactions. Balanced random forest is retained across tasks for consistency.

### Experimental Setup

#### Datasets

We use the MIMIC-III dataset [38], which includes structured and unstructured data for over 40,000 intensive care unit patients. It includes structured data (demographics, vitals, laboratories, admissions, and *ICD-9 Clinical Modification* codes) and unstructured clinical text (physician notes, discharge summaries, and radiology reports). For this study, we focus on physician-authored notes containing clinical reasoning, assessments, and treatment plans. Here, we exclude discharge summaries and notes written after outcomes to prevent label leakage; otherwise, consistent with prior methods on MIMIC-III, we use the remaining structured and unstructured data recorded during the admission. Only 0*.*85% (1202/141,624) of notes mention hospice, indicating rare explicit terminal indicators. However, a limitation of MIMIC-III for the readmission task is the inability to distinguish planned or elective readmissions and interfacility transfers, which may inflate the count of “avoidable” readmissions.

#### Biomedical Knowledge

We use abstracts from the annual PubMed Baseline dataset, comprising over 36 million biomedical citation records, to build a medical KG that enriches LLM input and reduces hallucinations. We also incorporate UMLS [28] to construct concept-centric subgraphs from EHR data.

#### Dataset Statistics

We include a summary table in Multimedia Appendix 1, showing dataset statistics, indicating moderate class imbalance for in-hospital mortality (~13% positive) and severe imbalance for 30-day readmission. To prevent data leakage, train-test splits (80:20) are patient disjoint, meaning that multiple visits from the same patient do not appear in both sets.

#### Baselines

We compare against Claude 3.7 Sonnet [39], MedGemma [40], LLaMA3-Med [41], and KARE [15], as well as structured-data models including logistic regression, tree-based models, and MLPs used in prior work on MIMIC-III [42-44]. All LLMs are evaluated in a zero-shot setting with the same patient-context prompt. KARE uses a similar patient retrieval but lacks clinical notes. Our model incorporates retrieved notes for better context. Implementation details are in Multimedia Appendix 1.

#### Metrics

We evaluate model performance using a comprehensive set of measures. Area under the receiver operating characteristic curve (AUROC) and area under the precision-recall curve (AUPRC) capture discrimination ability across thresholds, while overall accuracy reflects the proportion of correct predictions. For class-specific evaluation, we report precision (positive predictive value), recall (sensitivity), negative predictive value (NPV), and specificity. These capture both the model’s ability to correctly identify positive cases (sensitivity and positive predictive value) and its reliability on negatives (specificity and NPV). Finally, macro *F*_1_-score balances precision and recall across classes, ensuring that performance on the minority positive class is not overshadowed by the majority class. All metrics are computed on a held-out test set using standardized preprocessing to ensure comparability across models.

### Performance of KAMELEON

#### Overview

We explore different kinds of standard ML methods in *M*_2_ for making the final prediction, using all the integrated inputs. We refer to the corresponding algorithm as KAMELEON-X, where X represents Balanced-RF (balanced random forest), logistic regression, random forests, long short-term memory, LightGBM, MLP, or XGBoost.

#### 30-Day Readmission Prediction

##### Overview

Readmission within 30 days is a highly imbalanced task, with only about 4% (403/10,031) positive cases in the dataset. This severe imbalance is reflected in the results, where most models achieve high accuracy and precision on the negative class but struggle with recall for the positive (readmitted) class.

As shown in Table 3, our framework with a balanced random forest (KAMELEON-BalancedRF) classifier achieves the highest AUROC (0*.*845) and notably improves recall on positive cases to 0*.*79, a crucial metric since identifying patients at risk of readmission is clinically imperative. The KAMELEON-MLP model, while achieving the highest overall accuracy (0*.*91) and macro *F*_1_ (0*.*58), still attains a sensitivity of 0*.*28 on positive cases, illustrating the persistent challenge in detecting rare events. Unstructured LLM-based baselines such as Claude-3.7-Sonnet, MedGemma, LLaMA3-Med, and KARE show substantially lower sensitivity for positives (below 0.3), suggesting that these models struggle to identify the minority class without further fine-tuning or domain-specific adaptation.

In Table 3, KAMELEON-BalancedRF achieves a precision of 0.13, meaning that about 1 in 8 patients flagged as high-risk were actually readmitted. Recall (sensitivity) is 0.79, indicating that the model correctly identifies nearly 8 out of 10 true readmissions—a clinically critical result. The *F*_1_-score of 0.55 reflects the balance between precision and recall. The NPV is 0.99, showing that almost all patients predicted as low risk were indeed not readmitted. Specificity is 0.80, meaning the model correctly classifies 8 in 10 patients who were not readmitted as low risk.

**Figure 3:**
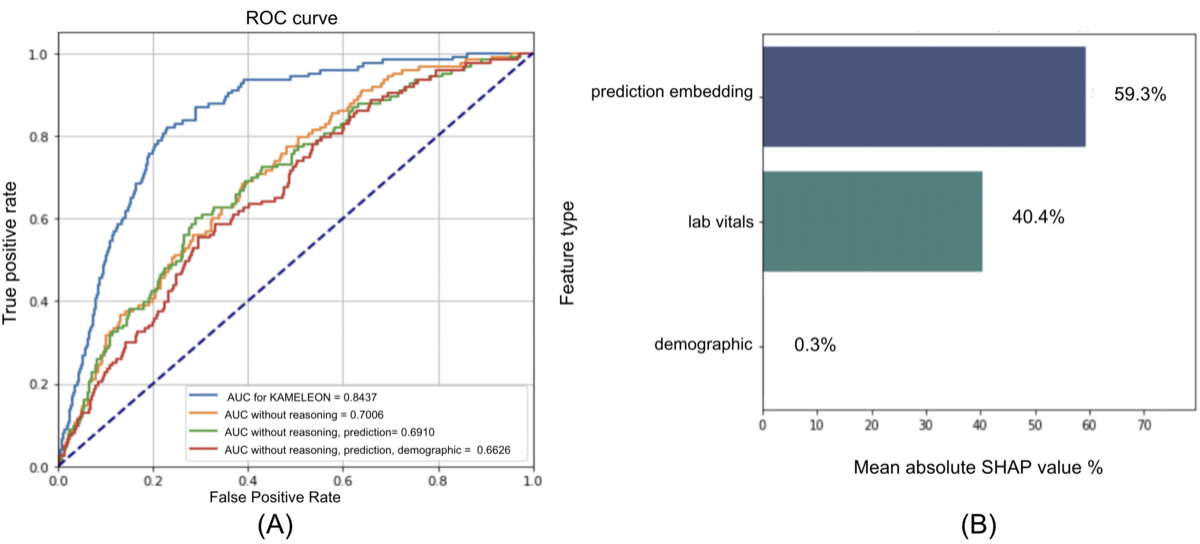
(A) KAMELEON (Knowledge-Augmented Multimodal EHR Learning for Outcome Prediction) achieves the highest area under the curve (AUC) for 30-day readmission when combining all features of unstructured model (M1) and structured model (M2), outperforming ablation variants. (B) Shapley additive explanations (SHAP) analysis shows prediction embeddings from M1 are key contributors.

To better understand this model’s behavior, we perform Shapley additive explanations (SHAP) analysis to identify feature importance, and Figure 3 indicates that the model relies primarily on prediction embeddings (59*.*3%) and laboratory/vital features (40*.*4%) for predicting 30-day readmission, highlighting the importance of multimodal inputs.

While prior studies like Morgan et al [45] reported AUROCs up to 0.81 for readmission, and general models typically ranged from 0.61 to 0.73 [46,47], our multimodal approach effectively captures complex clinical nuances.

**Table 3:** Comparison of models for 30-day readmission and in-hospital mortality prediction. Scores from Knowledge Aware Reasoning-Enhanced HealthCare Prediction (KARE) [15] are reevaluated using our pipeline due to large language model differences and incorrect data preprocessing in their code. Reported metrics include accuracy, negative predictive value (NPV), precision (positive predictive value), sensitivity (recall), specificity, macro F1, area under the receiver operating characteristic curve (AUROC), and area under the precision-recall curve (AUPRC).

Model			_*D*_ *struct* ^a^		_*D*_ *unstruct* ^b^		Accuracy		NPV		Precision		Specificity		Sensitivity		Macro *F*_1_		AUROC		AUPRC
**Task:** **30-day** **readmission** **prediction**																					
	Logistic regression [42]	✓				0.831		0.869		0.036		0.951		0.013		0.464		0.463		0.090	
	MLP^c^ [29]	✓				0.828		0.876		0.182		0.934		0.100		0.516		0.559		0.165	
	BalancedRF^d^	✓				0.760		0.970		0.070		0.780		0.430		0.490		0.673		0.066	
	LSTM^e^ [42]	✓				0.820		0.876		0.163		0.925		0.100		0.512		0.569		0.152	
	Claude-3.7-Sonnet^f^ [39]			✓		0.240		0.790		0.199		0.068		0.927		0.227		0.498		0.199	
	MedGemma-4b-it^f^ [40]			✓		0.350		0.770		0.190		0.270		0.690		0.350		0.480		0.190	
	LLaMA3-Med42-8B^f^ [41]			✓		0.390		0.800		0.210		0.360		0.670		0.410		0.510		0.210^g^	
	*M*_1_^h^			✓		0.660		0.870		0.130		0.720		0.280		0.480		0.506		0.195	
	KARE [15]			✓		0.271		0.785		0.191		0.131		0.851		0.269		0.491		0.191	
	KAMELEON^i^-LogReg^j^, (C=1.0)	✓		✓		0.833		0.869		0.037		0.953		0.013		0.519		0.130		0.148	
	KAMELEON-LogReg, (C=0.01)	✓		✓		0.871		0.874		0.333^g^		0.996^g^		0.013		0.478		0.551		0.152	
	KAMELEON-LSTM	✓		✓		0.840		0.880		0.190		0.950		0.090		0.510		0.505		0.135	
	KAMELEON-BalancedRF	✓		✓		0.800		0.990^g^		0.130		0.800		0.790^g^		0.550		0.845^g^		0.150	
	KAMELEON-MLP	✓		✓		0.910^g^		0.970		0.160		0.940		0.280		0.580^g^		0.820		0.138	
**Task:** **in-hospital** **mortality** **prediction**																					
	Logistic regression [42]	✓				0.850		0.912		0.340		0.916		0.331		0.625		0.624		0.190	
	LSTM [42]	✓				0.690		0.800		0.260		0.800		0.250		0.530		0.560		0.240	
	BalancedRF	✓				0.810		0.950		0.340		0.820		0.700		0.670		0.860		0.475	
	LightGBM^k^ [29]	✓				0.890		0.930		0.510		0.940		0.480		0.720		0.866		0.534	
	MLP [29]	✓				0.870		0.920		0.430		0.920		0.430		0.680		0.829		0.426	
	XGBoost^l^	✓				0.890		0.920		0.520		0.950		0.380		0.695		0.835		0.487	
	Claude-3.7-Sonnet^f^ [39]			✓		0.800		0.890		0.120		0.880		0.130		0.510		0.510		0.110	
	MedGemma-4b-it^f^ [40]			✓		0.120		0.950		0.100		0.020		0.990		0.120		0.510		0.110	
	LLaMA3-Med42-8B^f^ [41]			✓		0.160		0.950		0.120		0.100		0.970		0.190		0.530		0.120	
	*M*_1_			✓		0.614		0.890		0.134		0.641		0.413		0.474		0.527		0.125	
	KARE [15]			✓		0.639		0.885		0.129		0.678		0.353		0.478		0.515		0.122	
	KAMELEON-BalancedRF	✓		✓		0.880		0.930		0.490		0.934		0.492		0.710		0.876		0.543	
	KAMELEON-LSTM	✓		✓		0.730		0.820		0.430		0.840		0.390		0.620		0.740		0.350	
	KAMELEON-LightGBM	✓		✓		0.880		0.940^g^		0.470		0.910		0.590^g^		0.730		0.890		0.550	
	KAMELEON-MLP	✓		✓		0.900		0.940^g^		0.550		0.940		0.550		0.750^g^		0.890		0.600	
	KAMELEON-XGBoost	✓		✓		0.920^g^		0.920		0.790^g^		0.980^g^		0.369		0.660		0.920^g^		0.650^g^	

^a^*D_struct_*: structured data.

^b^*D_unstruct_*: unstructured data.

^c^MLP: multilayer perceptron.

^d^BalancedRF: balanced random forests.

^e^LSTM: long short-term memory.

^f^Models are evaluated in a zero-shot setting without fine-tuning.

^g^Best-performing value.

^h^*M*_1_: unstructured data encoder.

^i^KAMELEON: Knowledge-Augmented Multimodal EHR Learning for Outcome Prediction.

^j^LogReg: logistic regression.

^k^LightGBM: light gradient boosting machine.

^l^XGBoost: extreme gradient boosting.

##### Relative Importance of Different Classes of Inputs

We conduct an ablation study for the readmission task, where we retrain KAMELEON after dropping different components—the retraining and prediction models from *M*_1_ and the demographics used in *M*_2_ (Figure 3 and Table 4). We find that the reasoning component output by *M*_1_ is very significant and affects multiple metrics beyond AUROC. In the full model, KAMELEON achieves balanced performance (accuracy=0.80; macro *F*_1_=0.55; AUROC=0.844; AUPRC=0.147) with both high specificity (0.80) and sensitivity (0.77). When we drop the reasoning component, sensitivity falls by over 80% (falling to 0.06), and AUPRC is nearly halved, revealing strong bias toward the majority class; however, there is a gain in accuracy (rising to 0.92). Removing the reasoning component from KAMELEON drops performance from 0.844 to 0.7, a 17% decline in AUROC, highlighting the critical role of the fine-tuned LLM’s reasoning in risk prediction. Removing reasoning and prediction (output from *M*_1_) causes intermediate degradation, with AUROC falling by ~18%, consistent with the loss of calibrated probability signals and semantic rationale. Eliminating reasoning together with demographics and prediction yields the sharpest overall decline, with macro *F*_1_ dropping by ~13% and AUROC by more than ~22% (to 0.663), confirming the complementary value of these components. These analyses collectively demonstrate that reasoning substantially improves minority-class detection: it boosts sensitivity and AUPRC by more than threefold compared to variants without it, while also preventing misleading accuracy gains driven solely by the dominant negative class. Figure 3 visualizes these contributions, showing that reasoning, *M*_1_’s prediction embedding, and demographics each add critical and nonredundant signals for accurate readmission risk estimation.

**Table 4:** Ablation study on readmission.

Model	Accuracy	NPV^a^	Precision (PPV^b^)	Specificity	Sensitivity	Macro *F*_1_	AUROC^c^	AUPRC^d^
KAMELEON^e^	0.80	0.99	0.13	0.80	0.77	0.55	0.844	0.147
Without reasoning_*M1*_	0.94	0.96	0.07	0.97	0.06	0.52	0.699	0.078
Without demographic, reasoning_*M1*_, prediction_*M1*_	0.73	0.97	0.06	0.74	0.45	0.48	0.663	0.061
Without reasoning_*M1*_, prediction_*M1*_	0.74	0.97	0.09	0.75	0.55	0.50	0.691	0.062

^a^NPV: negative predictive value.

^b^PPV: positive predictive value.

^c^AUROC: area under the receiver operating characteristic curve.

^d^AUPRC: area under the precision-recall curve.

^e^KAMELEON: Knowledge-Augmented Multimodal EHR Learning for Outcome Prediction.

##### Sample Inference Output From M1

Textbox 1 shows a sample output generated by the fine-tuned LLM for the 30-day hospital readmission prediction task. Instead of returning a raw binary value (0 or 1), the model is prompted to generate both a prediction and its reasoning based on the patient’s diagnoses, procedures, medications, and comparisons with similar cases. This structured explanation allows the model to ground its prediction in a clinical context, improving reliability and interpretability over naive classification. By incorporating rationale into the output, the LLM demonstrates better alignment with real-world clinical decision-making.

**Inference results from a fine-tuned LLM for 30-day readmission prediction.**

**Patient ID: 25070 0**

To predict whether Patient ID 25070 0 will be readmitted within 30 days, we analyze the provided EHR context, focusing on conditions, procedures, and medications.

1. Conditions: The patient has multiple serious conditions, including non-Hodgkin’s lymphoma and complications from a device, which are significant risk factors for readmission. However, they also have other conditions that may not be as acute (eg, asthma and thyroid disorders).
2. Procedures: The patient has undergone several diagnostic procedures, including mastoidectomy, which could lead to complications but are not major surgeries. The patient did not have any major surgical interventions that typically increase readmission risk.
3. Medications: The patient is on a complex regimen including corticosteroids and other systemic medications. The presence of corticosteroids indicates a potential for complications, particularly in the management of their non-Hodgkin’s lymphoma.
4. Similar Patients: The similar patients show that those with chronic obstructive pulmonary disease and heart failure have higher readmission rates. However, Patient ID 25070 0 does not share the same chronic conditions with these patients.
5. Overall Assessment: Although the patient has several chronic and acute conditions, they do not have the same severity or complexity as the patients who have been readmitted. The absence of major recent surgeries and the complexity of their medication regimen suggest a lower risk of readmission.

**# Prediction #**

0

**Patient ID: 93900 1**

To predict whether Patient ID: 93900 1 will be readmitted to the hospital within 30 days, we analyze the patient’s EHR context, focusing on conditions, procedures, and medications.

1. Conditions: The patient has multiple chronic conditions, including congestive heart failure, diabetes mellitus with complications, and various gastrointestinal disorders. These conditions are known to increase the risk of exacerbations and complications, which are significant predictors of readmission.
2. Procedures: The patient has undergone significant procedures, such as debridement and upper gastrointestinal endoscopy. These procedures can lead to complications, necessitating close monitoring and follow-up care, which may contribute to readmission.
3. Medications: The patient is on a complex medication regimen, including beta-blockers and insulins. The complexity of this regimen can lead to adherence issues and potential side effects, which might complicate the patient’s health status and increase the likelihood of readmission.
4. Similar Patients: The similar patients, such as Patient ID: 16994 6, experienced complications from chronic diseases and were readmitted. This indicates a trend in patients with similar conditions and treatment regimens.
5. Overall Risk Assessment: The combination of chronic conditions, significant procedures, and complex medication regimens suggests a high risk of readmission due to potential exacerbations and complications.

Given this comprehensive analysis, it is reasonable to conclude that Patient ID: 93900 1 will likely be readmitted to the hospital within 30 days based on the factors outlined above.

##### Analysis of Distribution (Readmission)

**Figure 4:**
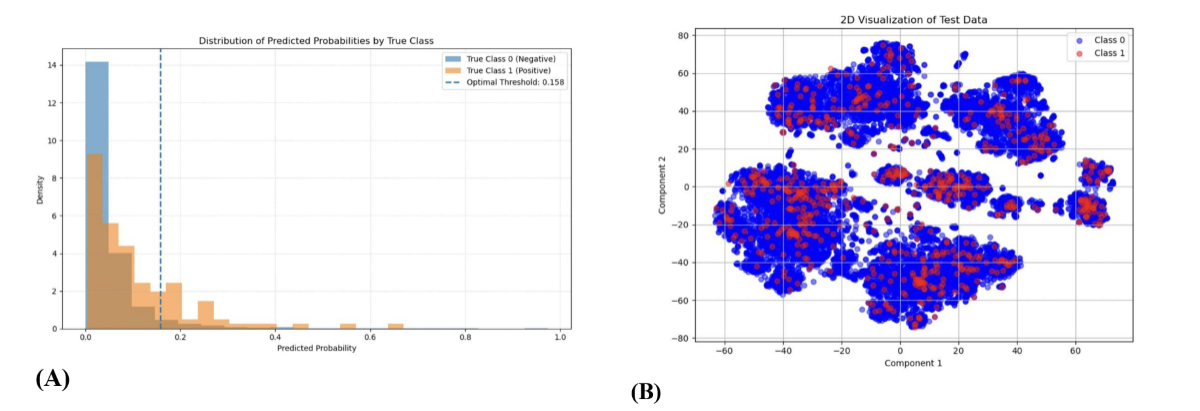
Visualization of class separability and predicted probability distributions for the readmission within 30 days. (A) Distribution of predicted probabilities for in-hospital mortality, separated by true class labels. The vertical dashed line marks the optimal threshold (0.276) balancing sensitivity and specificity. (B) T-distributed Stochastic Neighbor Embedding of the test dataset for the in-hospital mortality task, showing Class 0 (blue) and Class 1 (red).

Figure 4A illustrates the distribution of predicted probabilities for the readmission task. In the full model, the majority of negatives cluster near zero, while positives are shifted upward and concentrated above a low threshold of ~0.16. This optimal threshold is chosen using the Youden J statistic, which maximizes sensitivity and specificity. This separation, although not perfectly distinct, reflects the extreme class imbalance in the data: a very low cutoff is required to recover a reasonable fraction of positive cases. The overlap between classes explains the modest AUPRC values, since many positives still lie in regions dominated by negatives. Nevertheless, compared to ablated variants, the full model achieves tighter grouping of positives in the higher-probability region, resulting in better sensitivity and precision-recall trade-offs.

The t-distributed Stochastic Neighbor Embedding (t-SNE) plot (Figure 4B) further visualizes patients’ features on the held-out test set. In this space, readmitted patients do not form sharply separated clusters but instead appear partially embedded within the larger manifold of nonreadmitted cases. The lack of clear separation reflects the difficulty of the task and the subtlety of signals driving readmission, where positives and negatives overlap substantially. Still, there is evidence of localized groupings of readmitted patients, suggesting that the model captures some latent patterns that distinguish higher-risk subgroups. This partial clustering is consistent with the modest AUPRC values: while the model cannot fully disentangle the classes, it is able to concentrate a portion of true positives in regions of elevated probability. Clinically, this underscores the challenge of predicting readmission but also highlights the value of identifying even partially coherent patient subgroups for targeted follow-up.

#### In-Hospital Mortality Prediction

##### Overview

**Figure 5:**
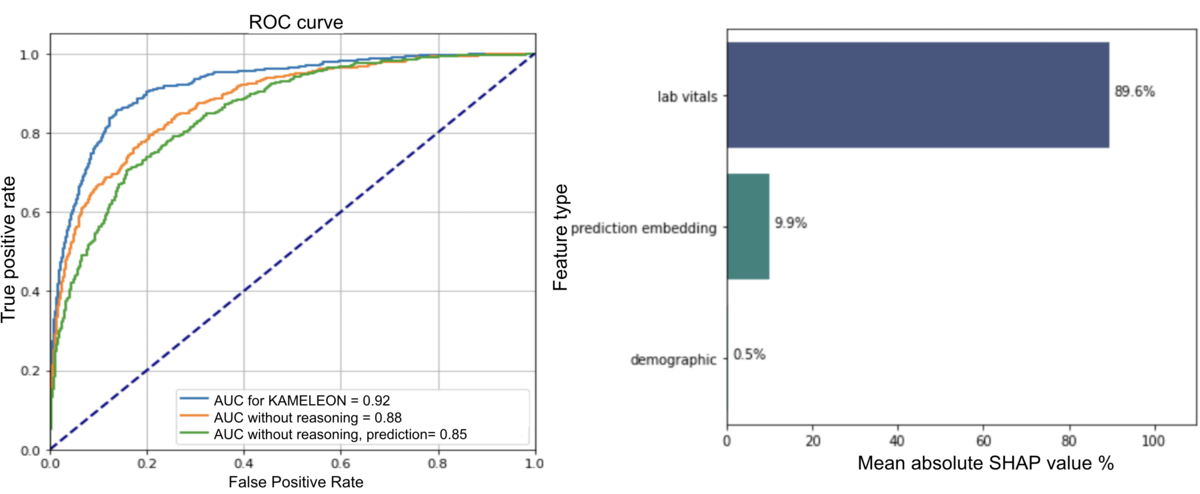
Performance and feature importance for the mortality prediction task. (A) Area under the curve (AUC) for the mortality prediction task. (B) Importance of features for predicting mortality. KAMELEON: Knowledge-Augmented Multimodal EHR Learning for Outcome Prediction; ROC: receiver operating characteristic curve; SHAP: Shapley additive explanations.

Mortality prediction is less imbalanced, with approximately 13% positive cases. Table 3 reports that KAMELEON-XGBoost and KAMELEON-MLP models achieve high accuracy (0*.*92 and 0*.*90, respectively) and AUROC (0*.*92 and 0*.*89, respectively). KAMELEON-XGBoost demonstrates strong performance for mortality prediction, achieving a high precision of 0*.*79, meaning that most patients flagged as high risk did not survive. It also attains a specificity of 0*.*98 and an NPV of 0*.*92, indicating that nearly all patients predicted as low risk were indeed survivors. Furthermore, for a positive class that, while less imbalanced, is still a minority, the AUPRC is a vital metric. Here, the KAMELEON with 0*.*650 sets the benchmark, significantly outperforming all other baselines. Unstructured models consistently yield the lowest performance for mortality prediction, AUROC values hover just above random chance (around 0*.*51-0*.*53), and AUPRCs remain very low (maximum 0*.*125), indicating a limited ability to discern between mortality and survival solely from clinical notes. SHAP results (Figure 5B) demonstrate that laboratory results and vital signs strongly drive predictions, with prediction embeddings playing a less critical role compared to readmission. Overall, Table 3 shows that for both tasks, our multimodal model outperforms all individual structured and unstructured baselines across all metrics.

##### Relative Importance of Different Classes of Inputs

The SHAP values (Figure 5B) show that the prediction embedding has a smaller influence compared to laboratories and vitals, which contrasts with the readmission task. The AUROC curve (Figure 5A) further illustrates that removing reasoning reduces AUROC from ~0.92 in the full model to ~0.88, a relative drop of about 4%. This indicates that mortality prediction is comparatively easier, as a strong AUROC is retained even without reasoning. The lower-class imbalance and the strong signal contained in laboratory values and diagnostic codes allow the model to capture patterns associated with terminal illness more directly. Nevertheless, reasoning still contributes by improving discrimination at the margin, capturing subtler risk factors that are less apparent in structured features alone.

##### Sample Inference Output From M1

We show inference outputs for 2 patients on the mortality prediction task, generated by *M*_1_, our fine-tuned LLM with reasoning, in Tables S10 and S11 in Multimedia Appendix 1.

##### Analysis of Distribution (In-Hospital Mortality)

**Figure 6:**
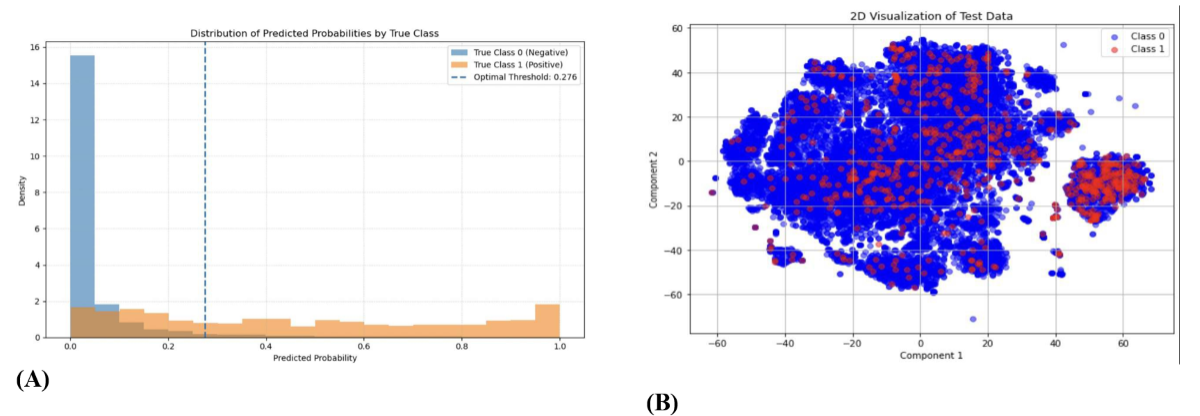
Visualization of class separability and predicted probability distributions for the in-hospital mortality task. (A) Distribution of predicted probabilities for in-hospital mortality, separated by true class labels. The vertical dashed line marks the optimal threshold (0.276) balancing sensitivity and specificity. (B) T-distributed Stochastic Neighbor Embedding of the test dataset for the in-hospital mortality task, showing Class 0 (blue) and Class 1 (red).

Figure 6A shows predicted probabilities for in-hospital mortality, separated by true class: survivors (blue) and deceased (red). The dashed line marks the optimal threshold (0.276) balancing sensitivity and specificity.

Most survivors cluster near zero probability, reflecting strong model confidence, while deceased cases spread across a wider range, showing prediction uncertainty. Overlap between classes causes some misclassifications, highlighting the challenge of predicting this rare event. Despite this, the clear separation and tight clustering of survivors demonstrate the model’s strong ability to distinguish between classes, supporting the usefulness of the selected threshold.

##### Class Separability Analysis Using t-SNE (In-Hospital Mortality Task)

Figure 6B shows a 2-dimensional t-SNE embedding of the test data, where the samples are colored by their true class labels (Class 0 in blue and Class 1 in red). The visualization reveals that the majority of the data forms a consistent, structured manifold dominated by Class 0 points, with only a small fraction of Class 1 points distributed across the embedding. Notably, a compact cluster of Class 1 samples appears on the right-hand side, indicating localized patterns that can be exploited by advanced models. This structure suggests that although Class 1 is relatively sparse, it exhibits distinct feature signatures in specific regions, which the proposed KAMELEON-X model is designed to capture effectively, contributing to improved predictive performance.

#### Incorporating Additional Patient Context in M1

Our unstructured model, *M*_1_, extends KARE [15] by incorporating physician notes more explicitly into similar patients, enriching the KG; in contrast, KARE [15] uses only structured EHR data related to drugs, procedures, and conditions as context for similar patients, and the KG is constructed without considering patient conditions. Our modification ensures that unstructured clinical narratives contribute to prediction alongside structured features, providing a stronger and more comparable baseline. Our strategy for adding physician notes leads to modest improvements in mortality and more substantial gains in readmission (Table 3). For mortality prediction, AUROC and AUPRC improve by ~2.3% and ~1.8%, respectively. For readmission, the gains are stronger, with AUROC improving by ~3.0% and AUPRC by ~2.1%. These results suggest that physician notes provide a useful complementary signal, especially for readmission, where unstructured narratives capture behavioral, discharge-related risk factors less visible in structured EHR data.

## Discussion

### Principal Findings

In this work, we introduce KAMELEON, a novel framework that effectively integrates multimodal EHR data, including structured clinical features and unstructured physician notes, enhanced by knowledge-augmented LLM reasoning, for robust clinical risk prediction. Our 2-stage architecture demonstrated superior performance on both 30-day readmission and in-hospital mortality prediction, with respect to multiple metrics, including the AUROC score. KAMELEON outperforms all prior baselines, which only used one type of dataset (structured or unstructured), on most metrics for these 2 tasks, compared to prior work using

the MIMIC-III dataset (Table 3). Multiple types of standard ML methods have been used for these tasks, yet KAMELEON demonstrates clear improvements across all evaluation metrics. None of the currently most powerful LLMs, including a medical LLM trained on clinical data, has comparable performance to KAMELEON. The only exception is that the LLaMA3-Med42-8B model achieves a higher AUPRC for the readmission task; however, KAMELEON significantly outperforms it across all other metrics.

The relatively lower performance of the LLM baselines reflects known limitations of standard LLMs in clinical prediction tasks. In our setup, LLM baselines receive only physician notes and therefore lack access to structured EHR signals such as laboratory values, vitals, and coded diagnoses that are critical for accurate risk prediction. At the same time, structured-only baselines (eg, XGBoost) also demonstrate limited performance, indicating that structured signals alone are insufficient. These results highlight the complementary strengths of structured and unstructured modalities and motivate the multimodal fusion design of KAMELEON, which integrates both sources while grounding reasoning with external biomedical knowledge.

We find that the reasoning component output by the LLM in *M*_1_, which is used in *M*_2_ by constructing an embedding, has high predictive power in both tasks. For the 30-day readmission task, the embedding constructed using the reasoning output by *M*_1_ is very significant—removing this component causes the AUROC to drop from 84*.*4% to 68*.*7%. This effect is much smaller in the case of the mortality prediction task, but not negligible, dropping the AUROC from 0.92 to 0.88 when this component is dropped. This highlights the synergy achieved by combining these diverse modalities.

This work underscores the significant potential of knowledge-augmented multimodal EHR modeling to enhance early intervention, optimize resource allocation, and improve patient care in complex clinical settings. While LLMs, including medical LLMs trained on specialized data, have a number of limitations in terms of accuracy and hallucinations, their reasoning outputs provide valuable predictive power.

Future work will focus on further validating KAMELEON’s generalizability across diverse clinical settings and exploring its application to a wider range of predictive health care tasks. Our framework can be easily extended to other clinical prediction tasks, especially those for which structured models have already been developed. KAMELEON can be applied for such tasks without any changes, and we expect it will provide similar gains.

### Scope and Promise for Social Impact

KAMELEON offers a strong opportunity to reduce avoidable hospital readmissions, a major driver of morbidity, cost, and financial penalties [48-51]. It provides real-time risk predictions for inpatients, enabling more effective discharge planning, case management, and postacute care. Our model uses real-time patient data to assess readmission risk prior to discharge, supporting individualized case management, discharge planning, census forecasting, and postacute care coordination. By identifying high-risk patients, our model enables focused use of limited resources, improving efficiency and outcomes.

## Appendix

Multimedia Appendix 1 Additional dataset details, framework explanation, result tables/plots.

## Abbreviations

AUPRC: area under the precision-recall curve
AUROC: area under the receiver operating characteristic curve
BalancedRF: balanced random forests
EHR: electronic health record
ICD: International Classification of Diseases
ICD-9: International Classification of Diseases, Ninth Revision
KAMELEON: Knowledge-Augmented Multimodal EHR Learning for Outcome Prediction
KARE: Knowledge Aware Reasoning-Enhanced HealthCare Prediction
KG: knowledge graph
LightGBM: light gradient boosting machine
LLM: large language model
M1: unstructured model
M2: structured model
MIMIC: Medical Information Mart for Intensive Care
ML: machine learning
MLP: multilayer perceptron
NPV: negative predictive value
SHAP: Shapley additive explanations
t-SNE: t-distributed Stochastic Neighbor Embedding
UMLS: Unified Medical Language System
XGBoost: extreme gradient boosting

## Data Availability

The study uses the open-access MIMIC-III database, which contains deidentified electronic health records. The dataset is available through PhysioNet [52] upon completing the required data use agreement and certification
