# Supplementary material for: Knowledge-Augmented Large Language Model for Multimodal Electronic Health Record–Based Risk Prediction: Development and Validation Study
Source: JMIR AI. 2026 Jun 12;5:e88356. doi: 10.2196/88356 (PMC13309766; doi:10.2196/88356)
Supplement: Multimedia Appendix 1 [file ai_v5i1e88356_app1.docx]

# Additional Details on Dataset

We use the Medical Information Mart for Intensive Care III (MIMIC-III) dataset [[35],](#_bookmark40) a large-scale, pub-licly available critical care database comprising deidentified health-related data associated with over 40*,* 000 patients who were admitted to the intensive care units (ICUs) of Beth Israel Deaconess Medical Center between 2001 and 2012. The dataset was curated to support clinical and epidemiological research and has since become a cornerstone resource in machine learning for healthcare.

MIMIC-III offers a comprehensive view of patient health trajectories by incorporating a diverse range of structured and unstructured data sources. Structured data include demographic information (e.g., age, gender, ethnicity), vital signs recorded at regular intervals (e.g., heart rate, blood pressure, respiratory rate, oxygen saturation), laboratory test results (e.g., glucose, pH, hematocrit), and hospital admission details (e.g., insurance type, admission location, length of stay, discharge disposition). Diagnosis and procedure codes are also available via ICD-9-CM, allowing for condition-based patient cohort extraction.

In addition to structured clinical variables, MIMIC-III contains extensive *unstructured clinical text*, such as physician notes, radiology reports, discharge summaries, Rehab Services, Echo, ECG , Case Manage-ment, General, Social Work, Pharmacy, Consult, and Nursing progress notes. These textual artifacts provide rich narrative context that captures clinical reasoning, impressions, and temporal evolution of patient condi-tions—information often not fully represented in structured fields.

For this study, we primarily focus on the unstructured physician notes that are authored during and after patient examinations. These notes include clinical assessments, differential diagnoses, treatment plans, and observations made by attending or resident physicians. We extract all available physician notes for each patient and temporally align them using the associated charted timestamps. To ensure semantic fidelity and medical relevance, a subset of the extracted notes is manually reviewed and annotated in collaboration with licensed physicians, enabling accurate downstream processing and interpretation by our model.

## Statistics of Dataset

The datasets for 30-day readmission and in-hospital mortality prediction are highly imbalanced, as only a small fraction of patients who are discharged are readmitted within 30 days. Table S[5](#_bookmark56) shows the percentage of positive cases for both tasks in the training and test datasets.

**Table S5:** Dataset statistics for mortality and readmission prediction tasks. Positive = target outcome occurred.

| **Task** | **Split** | **#Samples** | **% Positive** |
| --- | --- | --- | --- |

| In-Hospital Mortality | Train | 17903 | 13*.*53% |
| --- | --- | --- | --- |
|  | Test | 3236 | 11*.*55% |

| Readmission in 30 days | Train | 10031 | 4*.*01% |
| --- | --- | --- | --- |
|  | Test | 2425 | 3*.*80% |

## Features

Table S[6](#_bookmark57) outlines the structured and unstructured features used to train our framework. We incorporate all available patient, and visit-level information, including vitals, lab results, diagnosis and procedure codes, medications, and clinical notes, to provide a comprehensive representation of each patient’s condition. This multimodal input enables the model to learn both high-level clinical states and subtle variations across patients.

Table S[7](#_bookmark58) details the ICD-9 code groupings used to derive comorbidity indicators. These comorbidities are included as structured features, allowing the model to capture critical clinical context such as the presence of

chronic diseases (e.g., diabetes, cardiovascular disease) or acute conditions (e.g., sepsis, organ dysfunction). We also found that incorporating diagnosis type and the frequency of related conditions (e.g., number of cancer-related diagnoses) further improved prediction performance, suggesting that leveraging structured clinical coding hierarchies contributes meaningful signal to patient outcome modeling.

**Table S6:** Structured vs. Unstructured Features in the MIMIC-III Dataset

| **Type** | **Category** | **Examples** |
| --- | --- | --- |
| Structured | Static | Age, Gender, Ethnicity, Admission Type, Admission Location,  Insurance, Religion |
|  | Vitals | Heart Rate, Systolic/Diastolic/Mean Blood Pressure, Respiratory  Rate, Temperature, Oxygen Saturation, Capillary Refill Rate |
|  | Neurological Scores | Glascow Coma Scale: Eye Opening, Motor Response, Verbal Re-  sponse, Total |
|  | Lab Results | pH, Glucose, Fraction Inspired Oxygen |
|  | Physical Measures  Codes | Height, Weight  ICD-9 diagnosis codes, Procedure codes (CPT) |
| Unstructured | Clinical Notes | Discharge summaries, Progress notes, Nursing notes |
|  | Conditions  Procedures Medications | respiratory failure, complications of surgical procedures or med-  ical care, aspiration pneumonitis, etc  tracheostomy, respiratory, intubation and mechanical ventilation, gastrostomy etc  hypnotics, sedatives; drugs for constipation, other nutrients in  atc,adrenergics, inhalants, etc |

**Table S7:** ICD-9 Code Categories Used for Comorbidity Flags

| **Category** | **ICD-9 Prefixes (Examples)** |
| --- | --- |
| Explicit Sepsis | 99591, 99592, 78552, 038 |
| Infection | 001–139 (e.g., 001, 008, 038, 041, 079) |
| Organ Dysfunction | 584, 7855, 570, 572, 51881, 7991, 293 |
| Diabetes | 250 |
| Cardiovascular | 390–429 (e.g., 401, 410, 414, 427) |
| Cancer | 140–189 (e.g., 153, 162, 174, 185) |
| Lung Disease | 490–505 (e.g., 491, 493, 496, 500) |
| Dementia | 290, 294 |
| Kidney Dialysis | 585 |
| Liver Disease | 570, 571, 572 |
| Immune Disorder | 279 |

## Untructured Data Representation for *M*_1_

In the main paper, we described multiple stages of unstructured text encoder, *M*_1_. In this section, we will provide, examples on each step.


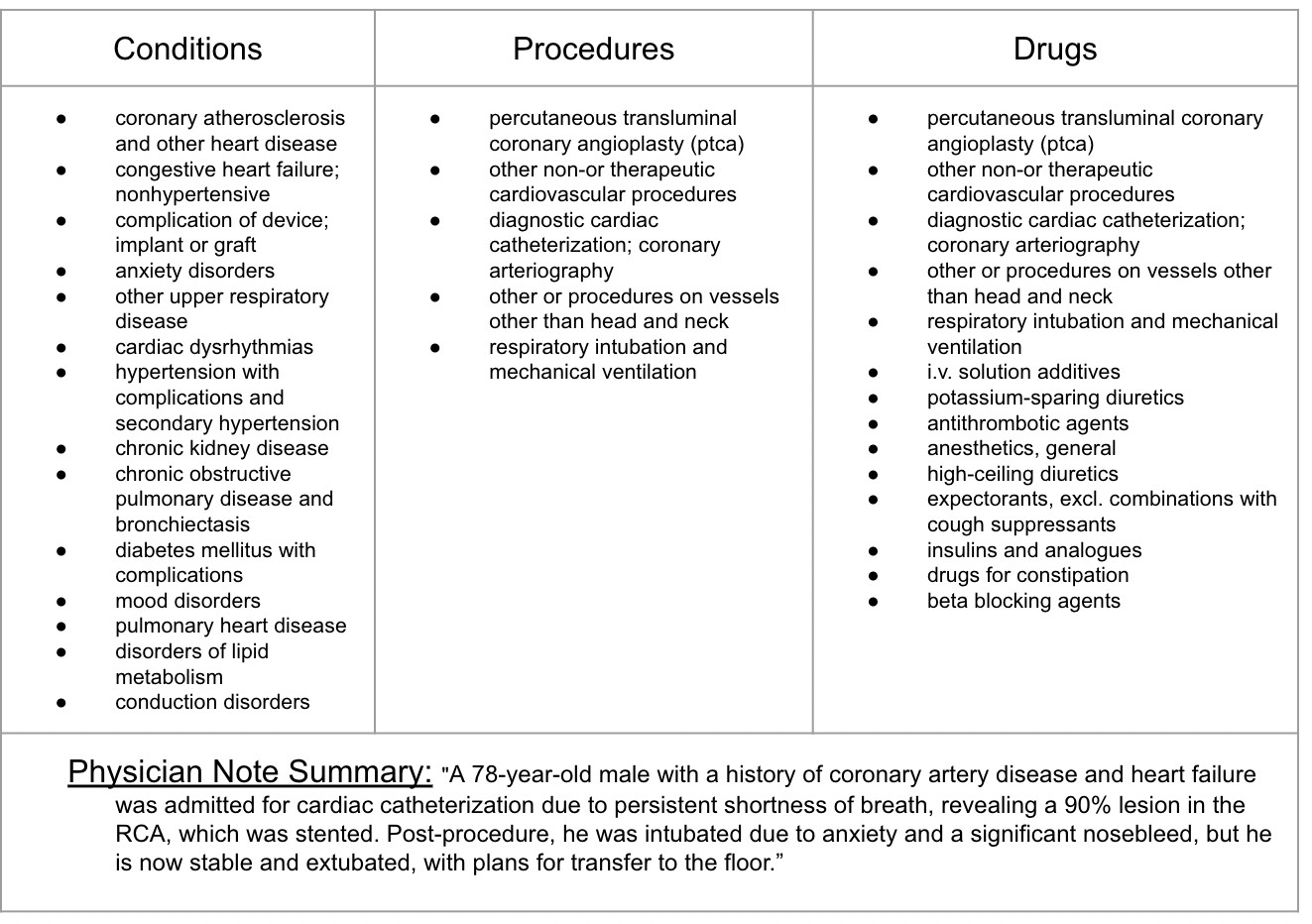


**Figure S6:** A sample generated context with physician summary for a single Patient Visit, for *M*_1_

### Generating Context

In this section, for each patient *P* ’s visit *v_i_*, we utilize information from the MIMIC-III dataset, including ICD codes and physician notes. These are used to extract the patient’s conditions, procedures, and prescribed drugs. Note that not all visits contain physician notes; in such cases, we leave the note field blank. If available, we include the note for the corresponding visit.

For patients with multiple visits, we aggregate information from all prior visits. This means a single entry may contain multiple physician notes concatenated together. However, these notes are often unstructured and may include irrelevant or redundant content, which can lead to context length issues when used as input to large language models (LLMs) during fine-tuning.

To address this, we employ a separate LLM to summarize the concatenated physician notes, producing a concise and clinically relevant summary for each patient history. Figure S[6](#_bookmark60) demonstrates a sample example of a patient context.

### Knowledge Graph Retrieval

The first step in our approach involves downloading the latest PubMed dataset and extracting all available abstracts. The goal is to capture general medical knowledge that may support diagnostic reasoning in com-plex clinical cases.

- - - - **Concept Generation**: After constructing the base context from these abstracts, we treat extracted medical conditions, procedures, and other relevant entities as concepts. In addition to the KARE

framework, we further enhance concept extraction from patients’ physician notes by applying a ded-icated NLP model to parse physician notes and extract clinical keywords and concepts directly from unstructured text. These are extracted using a biomedical natural language processing pipeline built on *scispaCy*. Each note is processed with the *en core sci sm* model to identify entities such as diseases, medications, procedures, symptoms, and conditions. Recognized terms are normalized to lowercase and stored as concepts. For notes where specific biomedical entities are not labeled, relevant noun phrases (longer than three characters) are also included to ensure broader coverage of meaning-ful medical concepts. These extracted concepts are later combined with structured EHR codes for downstream analysis.

- - - - **KG Construction**: Using these concepts, we perform targeted retrieval from the PubMed abstracts, identifying paragraphs that are semantically aligned with each concept. Once relevant text is retrieved, we employ a large language model (LLM) to extract relational triples (subject, predicate, object), thereby constructing a knowledge graph grounded in biomedical literature. Figure S[7](#_bookmark61) shows a partial snapshot of a knowledge graph constructed from PubMed data, filtered to include only patient-related concepts.
      - **KG structure**: This PubMed-derived knowledge graph supplements patient-specific data and im-proves reasoning by providing broader clinical context. The following triples are an example how the relations are extracted, involving *beta blocking agents* from biomedical literature.
        - (unstable angina pectoris, treated with, **beta blocking agents**)
        - **beta blocking agents**, should be avoided in, non responsive patients)
        - (**beta blocking agents**, could be a useful measure in, patients with labile arterial hypertension)
        - (**beta blocking agents**, could be a useful measure in, atients with vegetative dysregulation)
        - (**beta blocking agents**, could be a useful measure in, patients with hyperkinetic heart syndrome)
        - (bunitrolol, is a type of, **beta blocking agents**)
        - (**beta blocking agents**, impact, myocardial lactate extraction)
        - (**beta blocking agents**, reduce, arterial NEFA levels)

### Finding Similar Patients

Here, the model identifies similar patients by leveraging diagnoses, procedures, and medications, along with unstructured data from physician notes. For each patient visit, KARE extracts key clinical concepts and compares them across the cohort to find patients with overlapping medical histories and treatments. This similarity assessment enables more personalized predictions by grounding the model in relevant patient contexts.

### Reasoning Module

A critical component of our framework is the reasoning module, which enables the model to go beyond surface-level pattern matching and perform clinically informed inference. This module integrates the patient data—including summarized clinical notes, and the retrieved biomedical knowledge graph—to generate logical, interpretable explanations supporting the risk predictions.

Specifically, the reasoning module processes extracted clinical concepts and relationships from patient history and external knowledge to construct chains of evidence, mimicking clinician decision-making steps. This approach helps the model distinguish relevant signals from noise in complex and heterogeneous data.


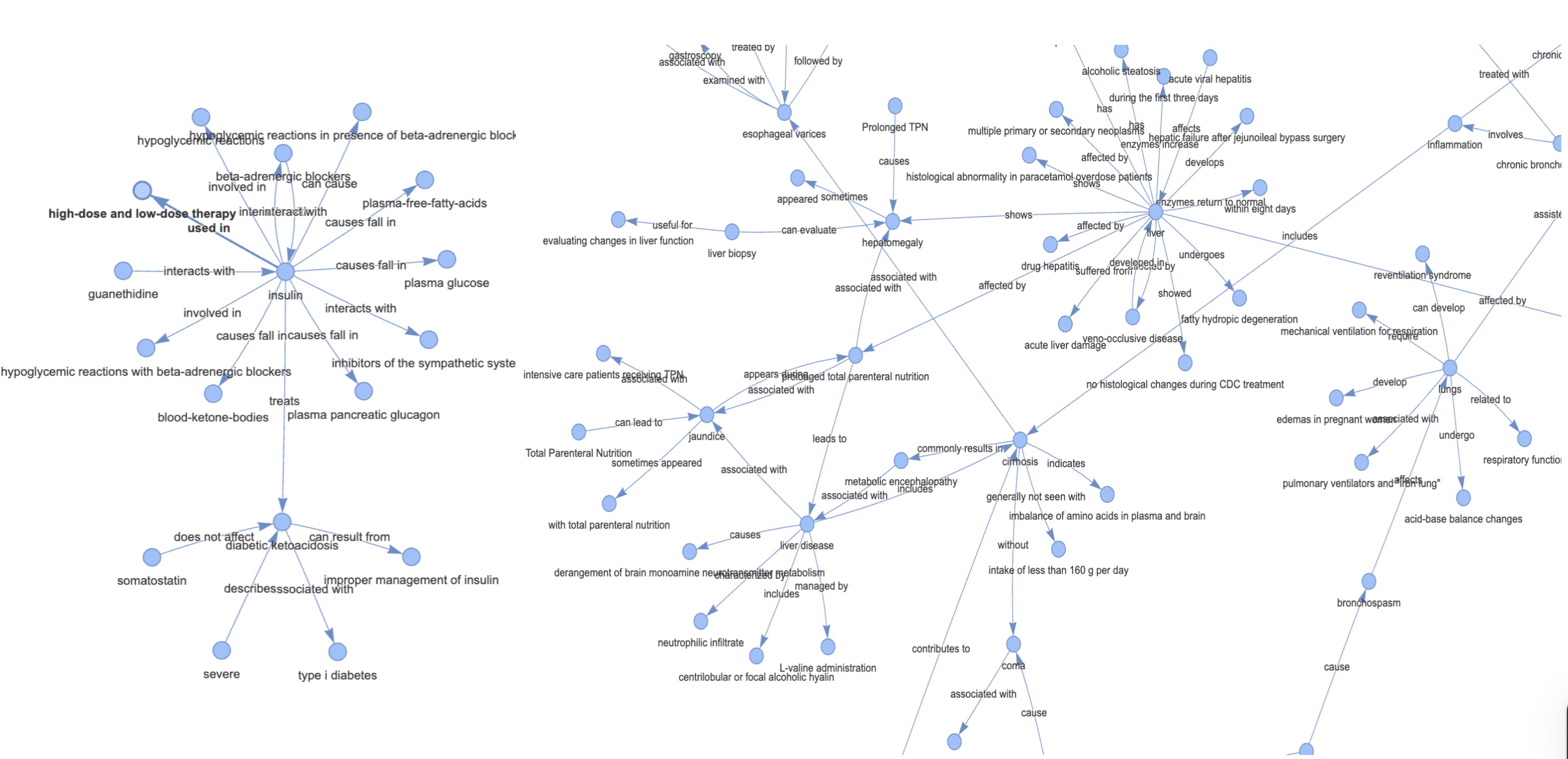


**Figure S7:** A partial snapshot of a knowledge graph built from PubMed data, filtered to include only patient-related concepts.


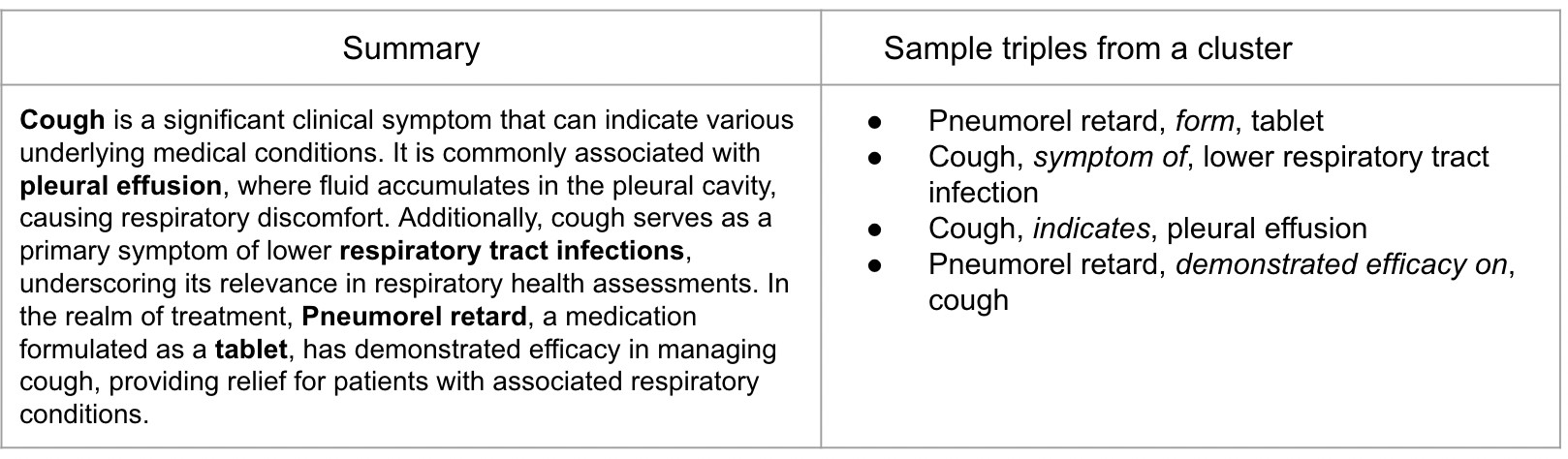


**Figure S8:** A cluster from the Knowledge Graph generated using the Leiden clustering algorithm, along with the corresponding LLM-generated summary of its triples.

**Table S8:** Example of Similar Patient Visits (both positive and negative) and Clinical Features for readmission in 30 days case

| **Feature** | **Patient ID: 12081 1, Visit 0** | **Patient ID: 12659 0, Visit 0** |
| --- | --- | --- |
| **Conditions** | Congestive heart failure, acute cerebrovas-  cular disease, respiratory failure, COPD, cardiac dysrhythmias, coronary atheroscle-rosis, conduction disorders | Benign neoplasm, heart valve disorders,  hepatitis, hypertension, lipid metabolism disorders |
| **Procedures** | Pacemaker insertion/revision, blood trans-  fusion, cardiac rhythm conversion | Heart procedures, extracorporeal circula-  tion, blood transfusion |
| **Medications** | ACE inhibitors, adrenergics (inhalants),  corticosteroids, lipid modifiers, cardiac stimulants, potassium supplements, an-tithrombotics, opioid analgesics | Antiinfectives, ACE inhibitors, beta block-  ers, calcium channel blockers, diuretics, analgesics, anxiolytics |
| **Physician**  **Notes** | Continued respiratory failure, CHF,  COPD; new lung cancer, heart valve disorders, diagnostic bronchoscopy, new medications | Not available |
| **Label** | 1 | 0 |

In Figure S[9,](#_bookmark64) the reasoning module is illustrated as a dedicated section within the overall prompt structure. It explicitly asks the LLM to produce detailed, medically grounded reasoning before outputting the final risk prediction, which improves both accuracy and interpretability. Ablation studies (Table S[2)](#_bookmark4) demonstrate that removing this reasoning component significantly degrades predictive performance, underscoring its essential role.

## Full Example Prompt

Figure S[9](#_bookmark64) illustrates the complete prompt structure used to fine-tune the LLM. The prompt includes **(1)** a clear task description; **(2)** the target patient’s electronic health record (EHR) context combining structured features and summarized clinical notes; **(3)** relevant external biomedical knowledge retrieved from PubMed abstracts represented as a knowledge graph; **(4)** examples of similar and dissimilar patient cases for comparative context (We show this in Table S[8;](#_bookmark63) and **(5)** reasoning behind the prediction, generated from an LLM.

The prompt concludes with an explicit request for medically grounded reasoning followed by the final risk prediction. This structured input design facilitates interpretable and accurate model outputs.

## Structured Data Representation for *M*_2_

We preprocess raw patient records by extracting structured data from MIMIC-III and organizing it into well-defined prediction datasets. We align each patient to an admission based on predefined cohort criteria, for in-hospital mortality prediction, we use early admission data, typically features collected within the first 24 hours, to build static representations. These include demographic attributes (such as age, gender, and admission type), physiological measurements (vital signs like heart rate or blood pressure), lab test results, and interventions. All continuous variables are normalized and discretized to create time-series bins or summary statistics. For readmission prediction, we rely on discharge time data or early records filtered using exclusion criteria (e.g., patients under 18 or with missing data), and construct 30-day readmission labels by linking consecutive admissions.

To handle missing values, we apply simple imputation strategies to ensure a consistent feature space across all patients. Specifically, missing entries in time-series data are filled using forward-fill and backward-fill methods where possible. For variables still missing after temporal imputation, we use either zero imputa-


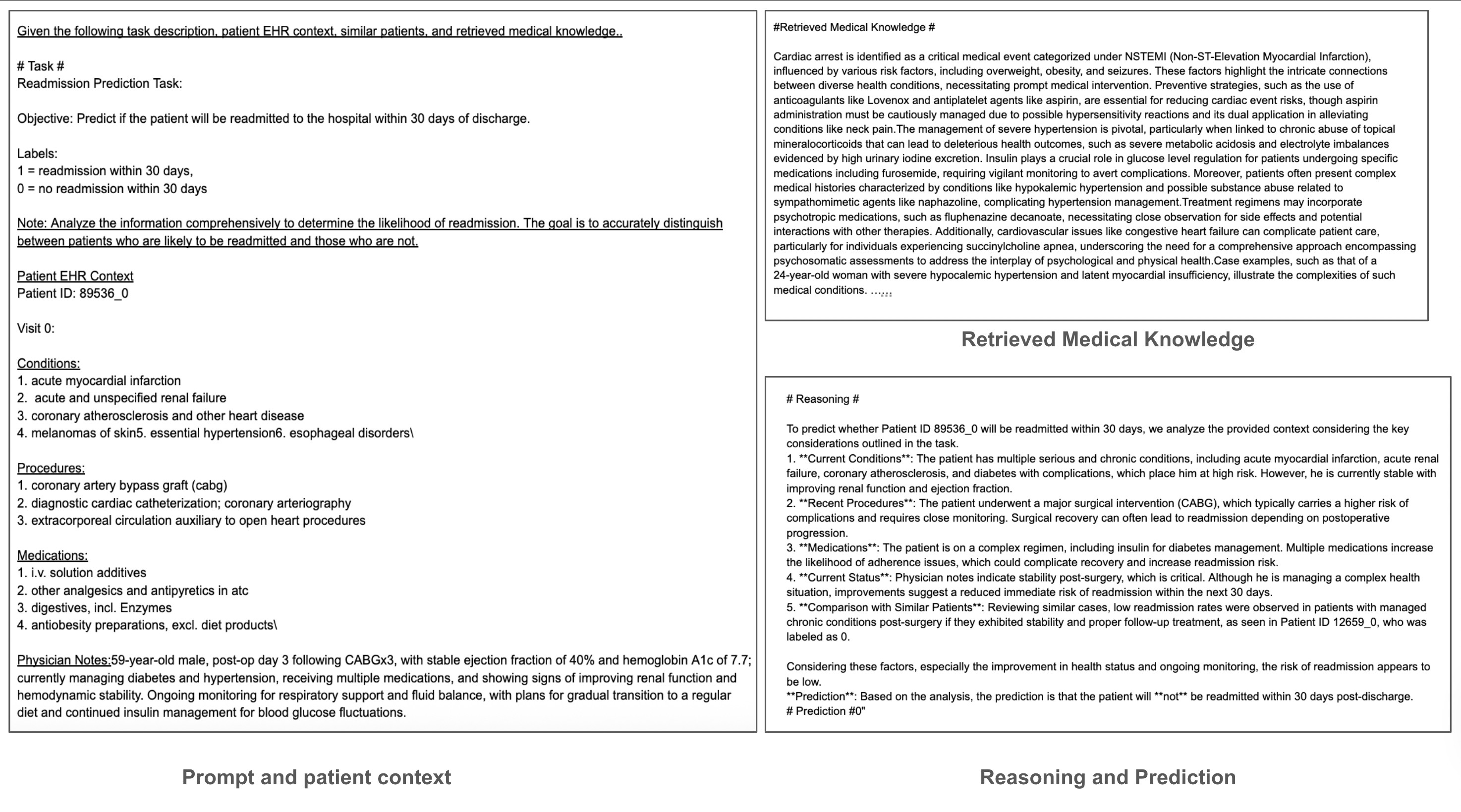


**Figure S9:** Example of the overall prompt structure provided to the LLM during fine-tuning, including task description, patient EHR context, retrieved medical knowledge, and expected reasoning and prediction outputs.

|  | **Positive** | **Negative** | **Anomalies Detected** | **Actual positive** |
| --- | --- | --- | --- | --- |
| Train Set | 718 | 17185 | 717 | 33 |
| Test Set | 123 | 3113 | 130 | 3 |

**Table S9:** Distribution of positive and negative samples across train, test, and anomaly detection settings using Isolation Forest method

tion (after normalization) or impute with population-level statistics such as the mean or median, depending on the variable type. This approach ensures that the downstream models receive complete input tensors without discarding any patient records due to sparsity, which is critical in clinical data settings where miss-ingness is common and often informative.

**Incorporating Patient Demographics and Admission Details** This step involves enriching the dataset with key patient demographics and hospital admission attributes. Then we merge external data tables containing information such as gender, ethnicity, admission type (emergency, elective), insurance, and language. Cate-gorical variables are converted into numeric codes for easier model consumption, while missing values are handled with placeholder categories to maintain data integrity. This results in a structured feature set that captures essential background and administrative details for each patient episode.

**Adding Clinical Diagnoses, Procedures, and Medication History** Next, the dataset is augmented by in-tegrating clinical history through diagnosis codes, procedure codes, and prescribed medications linked to each hospital admission. For instance, all ICD diagnosis codes recorded during a hospital stay are collected into a list, allowing the creation of binary flags that indicate the presence of major conditions like diabetes or cardiovascular disease. Additionally, common code prefixes are extracted to generate features represent-ing groups of related diagnoses or procedures. Similarly, medication records are summarized into key drug groups. This approach transforms complex clinical histories into a numerical format that models can use to assess patient risk.

**Embedding Clinical Reasoning and Merging Predictions** The final enrichment involves processing free-text clinical reasoning or explanations associated with predictions. These textual rationales are tokenized and converted into numerical vector embeddings that capture their semantic meaning, for example, repre-senting the sentence “patient shows signs of infection” as a 100-dimensional vector. These embeddings are paired with binary prediction labels and merged back into the main dataset by matching patient episodes. Missing values are filled with neutral defaults to ensure consistent data structure. The combined result is a dataset where each patient episode has both predictive labels and rich, encoded reasoning features to support improved downstream modeling or interpretation.

# Additional results

**Using Anomaly Detection Method for Readmission in 30 days**: Given the highly imbalanced nature of the 30-day readmission data, where only approximately 4% of hospital visits result in readmission, we explored whether this task could be framed as an anomaly detection problem. Specifically, we investigated whether the minority class (readmitted patients) exhibits anomalous patterns relative to the majority class.

To evaluate this, we applied unsupervised anomaly detection methods, such as the Isolation Forest algo-rithm, to identify readmissions as statistical outliers based on the available clinical features. Table S[9](#_bookmark65) shows the distribution of positive and negative samples in the training and test sets, along with the number of anomalies detected by the model.

Despite the appeal of this approach, anomaly detection methods did not perform well for this task. The results indicate that the readmitted patients do not form distinct statistical outliers in the feature space, mak-ing it difficult for unsupervised models to reliably separate them from the majority class. This suggests that the features available do not naturally differentiate readmissions as anomalous events, and that supervised learning approaches are more appropriate for this problem.

**Table S10:** Inference Results from Fine-Tuned LLM for Mortality Prediction (Part 1)

**Patient ID: 9782 1**

### **Step 1: Analyzing Conditions**

1. **Septicemia (except in labor)** - This is a severe condition that indicates systemic infection, which can lead to sepsis and high mortality risk.
2. **Shock** - Indicates a critical state, often associated with poor outcomes.
3. **Pneumonia** - This can be serious, especially in the context of septicemia and shock, as it can complicate respiratory function.
4. **Schizophrenia and other psychotic disorders** - While this is a mental health condition, it does not directly impact mortality.
5. **Deficiency and other anemia** - This can complicate other conditions, especially in the context of infection or respiratory issues.
6. **Thyroid disorders** - Generally less critical unless there are acute complications. ### **Step 2: Analyzing Procedures**
7. **Other vascular catheterization; not heart** - This is an invasive procedure that can carry risks, especially in the context of the patient’s other conditions.
8. **No major surgeries or life-sustaining procedures** - The absence of life-sustaining interventions suggests that the patient may not be in immediate critical condition.

### **Step 3: Analyzing Medications**

1. **Antibiotics (quinolone antibacterials, other antibacterials)** - Indicate the treatment of infection, which is critical given the septicemia and pneumonia.
2. **Corticosteroids for systemic use** - Can be used in severe conditions but also carry risks, especially in the presence of infections.
3. **Opioid analgesics** - Often used for pain management, which may indicate severe underlying conditions.
4. **Cardiac stimulants** - Suggests potential heart issues.
5. **High-risk medications** - The use of multiple medications indicates a complex health status, which increases the risk of adverse effects and complications.

### **Step 4: Comparing with Similar Patients**

1. **Patient ID 15209 0** had conditions such as septicemia, respiratory failure, and shock, leading to a label of 0 (survival). This patient had similar severe conditions but also underwent extensive invasive procedures and had more acute conditions.
2. **Patient ID 28061 0** had coronary atherosclerosis and cardiac issues leading to a label of 1 (mortality). This patient had significant cardiovascular issues and underwent multiple invasive procedures.

### **Step 5: Integrating Information**

- - The combination of septicemia, shock, and pneumonia in Patient ID 9782 1 is extremely concerning, especially consid-ering the lack of major surgical interventions. The presence of multiple comorbidities (anemia, schizophrenia, and thyroid disorders) complicates the overall picture.
  - The complexity and severity of the patient’s conditions (particularly septicemia and shock) indicate a high risk of mortality, but the absence of life-sustaining procedures suggests some resilience.

### **Conclusion**

Given the severity and complexity of Patient ID 9782 1’s conditions, the presence of multiple high-risk medications, and the lack of immediate life-threatening interventions, the prediction leans towards survival (label 0). However, the critical nature of the patient’s conditions suggests that the risk of mortality is significant, and a careful monitoring plan is essential for managing the patient’s health.

### **Final Prediction**

- - **Prediction**: 0 (survival)

**Table S11:** Inference Results from Fine-Tuned LLM for Mortality Prediction (Part 2)

**Patient ID: 85258 1**

### **Step 1: Analyze Patient Conditions**

1. **Cancer of Esophagus**: This is a critical condition that is often associated with high mortality risk, particularly when combined with other severe conditions.
2. **Secondary Malignancies**: The presence of secondary malignancies indicates that the patient has advanced cancer, further increasing the risk of mortality.
3. **Pneumonia**: This is a severe infection that can lead to respiratory failure, especially in a patient with compromised respiratory function due to cancer.
4. **Respiratory Failure**: The patient has been diagnosed with respiratory failure, indicating a critical state that signif-icantly increases the risk of mortality.
5. **Septicemia**: This is a serious systemic infection that can lead to multi-organ failure, which is a key indicator of mortality.
6. **Deficiency Anemia**: This can complicate the patient’s overall health, particularly in the context of cancer and infection.
7. **Other Comorbidities**: The patient has several other comorbidities, including disorders of teeth and jaw, white blood cell diseases, and fluid and electrolyte disorders, which compound the risk of mortality.

### **Step 2: Evaluate Procedures**

1. **Tracheostomy**: This is a significant surgical intervention that indicates severe respiratory compromise and is often performed in critically ill patients.
2. **Mechanical Ventilation**: The patient is on mechanical ventilation, which is a life-sustaining measure typically reserved for critically ill patients.
3. **Blood Transfusions**: The need for multiple blood transfusions suggests significant anemia or other severe condi-tions that require urgent intervention.

### **Step 3: Assess Medications**

1. **Broad Spectrum Antibiotics**: The patient is receiving broad-spectrum antibiotics, which are often used in the presence of sepsis or severe infections.
2. **Corticosteroids**: The use of corticosteroids suggests a significant inflammatory response or severe underlying condition.
3. **Antipsychotics and Antidepressants**: These medications suggest a complex mental health component, which can complicate treatment and recovery.

### **Step 4: Compare with Similar Patients**

1. **Patient ID: 28061 0**: This patient had a history of coronary artery disease and complications from medical care, leading to a mortality label of 1. The presence of critical cardiac issues and multiple comorbidities mirrors the complexity of Patient ID: 85258 1.
2. **Patient ID: 14297 0**: This patient had several chronic conditions but ultimately had a label of 0, indicating that not all patients with complex conditions lead to mortality. The complexity of Patient ID: 85258 1, however, suggests a much higher risk.

### **Step 5: Synthesize Findings**

- - The combination of advanced cancer, severe infections (septicemia), respiratory failure, and invasive procedures (tra-cheostomy and mechanical ventilation) strongly indicates that the patient is in a critical state.
  - The presence of multiple comorbidities and the complexity of the medications suggest that the patient is in a high-risk category for mortality.

### **Conclusion**

Given the severity of the conditions, the invasive nature of procedures, and the complex medication regimen, the patient is at an extremely high risk of mortality. Therefore, the prediction for Patient ID: 85258 1 is:

**Prediction**: 1 (mortality)
